# New scaling relations to compute atom-in-material polarizabilities and dispersion coefficients: part 1. Theory and accuracy†

**DOI:** 10.1039/c9ra03003d

**Published:** 2019-06-19

**Authors:** Thomas A. Manz, Taoyi Chen, Daniel J. Cole, Nidia Gabaldon Limas, Benjamin Fiszbein

**Affiliations:** Department of Chemical & Materials Engineering, New Mexico State University Las Cruces New Mexico 88003-8001 USA tmanz@nmsu.edu; School of Natural and Environmental Sciences, Newcastle University Newcastle upon Tyne NE1 7RU UK

## Abstract

Polarizabilities and London dispersion forces are important to many chemical processes. Force fields for classical atomistic simulations can be constructed using atom-in-material polarizabilities and C_*n*_ (*n* = 6, 8, 9, 10…) dispersion coefficients. This article addresses the key question of how to efficiently assign these parameters to constituent atoms in a material so that properties of the whole material are better reproduced. We develop a new set of scaling laws and computational algorithms (called MCLF) to do this in an accurate and computationally efficient manner across diverse material types. We introduce a conduction limit upper bound and *m*-scaling to describe the different behaviors of surface and buried atoms. We validate MCLF by comparing results to high-level benchmarks for isolated neutral and charged atoms, diverse diatomic molecules, various polyatomic molecules (*e.g.*, polyacenes, fullerenes, and small organic and inorganic molecules), and dense solids (including metallic, covalent, and ionic). We also present results for the HIV reverse transcriptase enzyme complexed with an inhibitor molecule. MCLF provides the non-directionally screened polarizabilities required to construct force fields, the directionally-screened static polarizability tensor components and eigenvalues, and environmentally screened C_6_ coefficients. Overall, MCLF has improved accuracy compared to the TS-SCS method. For TS-SCS, we compared charge partitioning methods and show DDEC6 partitioning yields more accurate results than Hirshfeld partitioning. MCLF also gives approximations for C_8_, C_9_, and C_10_ dispersion coefficients and quantum Drude oscillator parameters. This method should find widespread applications to parameterize classical force fields and density functional theory (DFT) + dispersion methods.

## Abstract 2

A new method was developed to compute atom-in-material polarizabilities and dispersion coefficients for diverse material types.

## Graphical abstract

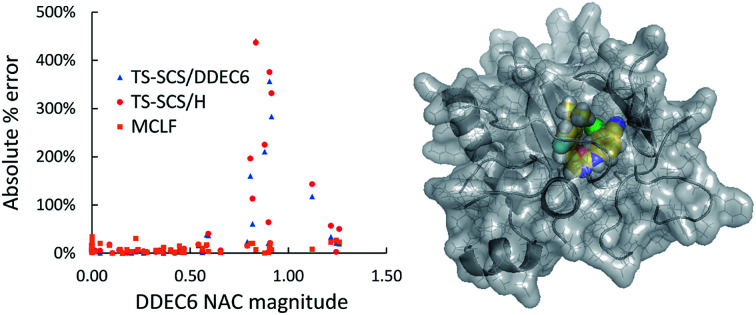

## 1. Introduction

When combined with large-scale density functional theory (DFT) calculations, the DDEC method has been shown to be suitable for assigning atom-centered point charges for flexible molecular mechanics force-field design.^1–8^ The assignment of C_6_ coefficients and atomic polarizabilities is another active area of research in force field design.^3,9–12^ Polarization effects are especially important for simulating materials containing ions.^13–20^ When considered alongside the importance of accurate theoretical methods to study van der Waals interactions at the nanoscale,^21^ it is clear that a crucial feature of new methods to compute these important quantities is the ability to scale to large system sizes in reasonable computational time.

In this article, we develop new scaling laws and an associated method to compute polarizabilities and dispersion coefficients for atoms-in-materials (AIMs). These new scaling laws and computational method give good results for isolated atoms, diatomic molecules, polyatomic molecules, nanostructured materials, solids, and other materials. This new method is abbreviated MCLF according to the authors' last name initials (where the C is both for Chen and Cole). We performed tests on isolated neutral and charged atoms, small molecules, fullerenes, polyatomic molecules, solids, and a large biomolecule with MCLF. The results were compared with experimental data, high level CCSD calculations, time-dependent DFT (TD-DFT) calculations, or published force-field parameters.

As discussed in several recent reviews and perspectives, the dispersion interaction is a long-range, non-local interaction caused by fluctuating multipoles between atoms in materials.^22–26^ It is especially important in (i) condensed phases including liquids, supercritical fluids, solids, and colloids, (ii) nanostructure binding such as the graphene layers forming graphite, and (iii) the formation of noble gas dimers. The dispersion interaction is closely related to AIM polarizabilities. The dispersion energy can be described by an expansion series. The leading term is inversely proportional to *R*^6^, where *R* is the distance between two atoms. The coefficient of this term is called the C_6_ dispersion coefficient, and this term quantifies fluctuating dipole–dipole interactions between two atoms. The intermolecular C_6_ coefficient is given by the sum of all interatomic contributions1
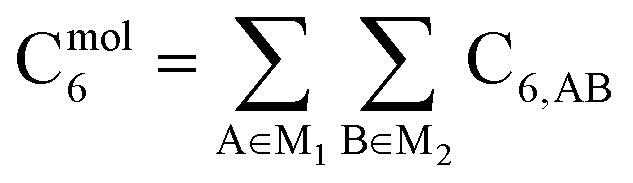
where M_1_ and M_2_ refer to the first and the second molecules. Higher-order terms represent different interactions.^27^ For example, the eighth-order (C_8_) term describes the fluctuating dipole–quadrupole interaction between two atoms. The ninth-order (C_9_) term describes the fluctuating dipole–dipole–dipole interaction between three atoms. The tenth-order (C_10_) term describes the fluctuating quadrupole–quadrupole and dipole–octupole interaction between two atoms.

Methods for computing polarizabilities and dispersion coefficients can be divided into two broad classes: (A) quantum chemistry methods that explicitly compute the system response to an electric field (*e.g.*, TD-DFT, CCSD perturbation response theory, *etc.*) and (B) AIM models. Class A methods can be highly accurate for computing polarizabilities and dispersion coefficients of a whole molecule, but they do not provide AIM properties. Therefore, class A methods cannot be regarded as more accurate versions of class B methods. Parameterizing a molecular mechanics force-field from quantum mechanics requires an AIM (*i.e.*, class B) model. Our goal here is not merely to develop a computationally cheaper method than TD-DFT or CCSD perturbation response theory to compute accurate system polarizabilities and dispersion coefficients, but rather to exceed the capabilities of both of those methods by providing accurate AIM properties for force-field parameterization.

One must distinguish the polarizability due to electron cloud distortion from the polarization due to molecular orientation (and other geometry changes) such as occurs in ferroelectric materials. Herein, we are solely interested in the polarizability that occurs due to electron cloud distortions. Electric polarization due to molecular orientation and other geometric changes can be described by a geometric quantum phase.^28–30^

There are several existing frameworks for calculating AIM polarizabilities and/or dispersion coefficients. Applequist *et al.*^31^ introduced a formalism that represents the molecular polarizability tensor in terms of AIM polarizabilities *via* the dipole interaction tensor. Thole^32^ refined this formalism by replacing atomic point dipoles with shape functions to avoid infinite interaction energies between adjacent atoms. Applequist's and Thole's methods use empirical atomic polarizability fitting to reproduce observed polarizability tensors of small molecules.^31,32^ Mayer *et al.* added charge–charge and dipole–charge interaction terms to calculate more accurate polarizabilities of conducting materials.^33,34^ Grimme *et al.* presented the D3 geometry-based method to calculate C_6_, C_8_, and C_9_ dispersion coefficients and dispersion energies, but this method does not yield polarizability tensors.^11^ The recently formulated D4 models are geometry-based methods that extend the D3 formalism by including atomic-charge dependence.^35–37^ The exchange-dipole model (XDM) is an orbital-dependent approach that yields AIM dipole, quadrupole, and octupole polarizabilities and C_6_, C_8_, and C_10_ dispersion coefficients.^38–42^ Several density-dependent XDM variants have been formulated.^43,44^ In 2009, Tkatchenko and Scheffler introduced the TS method for isotropic AIM polarizabilities and C_6_ coefficients.^12^ Both the XDM and TS methods yielded isotropic AIM polarizabilities rather than molecular polarizability tensors.^12,38–44^ In both the XDM and TS methods, the AIM unscreened polarizability is given by2
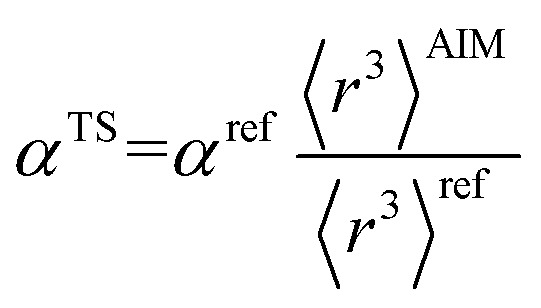
where “ref” refers to the reference value for an isolated neutral atom of the same chemical element, “AIM” refers to the partitioned atom-in-material value, and 〈*r*^3^〉 refers to the r-cubed radial moment. Both the XDM and TS methods originally used Hirshfeld^45^ partitioning to compute the 〈*r*^3^〉^AIM^ values.^12,42^

In 2012, Tkatchenko *et al.* introduced self-consistent screening (TS-SCS) *via* the dipole interaction tensor to yield the molecular polarizability tensor and screened C_6_ coefficients.^46^ The TS-SCS dipole interaction tensor uses a quantum harmonic oscillator (QHO) model similar to that used by Mayer but extended over imaginary frequencies and omitting charge–dipole and charge–charge terms.^33,34,46^ That same article also used a multibody dispersion (MBD^46,47^) energy model based on a coupled fluctuating dipole model (CFDM^47,48^). The TS-SCS screened static polarizability and characteristic frequency for each atom are fed into the CFDM model to obtain the MBD energy.^46^ The TS-SCS approach has advantages of yielding a molecular polarizability tensor and AIM screened polarizabilities and AIM screened C_6_ coefficients using only the system's electron density distribution as input.^46^

The TS-SCS method has several key limitations. Two key assumptions of the TS-SCS method are: (i) for a specific chemical element, the unscreened atomic polarizability is proportional to the atom's 〈*r*^3^〉 moment, and (ii) for a specific chemical element, the unscreened C_6_ coefficient is proportional to the atom's polarizability squared.^12,46^ However, in their work these hypotheses were not directly tested.^12,46^ Later, Gould tested these two assumptions and found them inaccurate for describing isolated neutral atoms placed in a confinement potential.^49^ Hirshfeld partitioning was used in the TS-SCS method^12,46^ to compute the 〈*r*^3^〉^AIM^. Because Hirshfeld partitioning uses isolated neutral atoms as references,^45^ the Hirshfeld method typically severely underestimates net atomic charge magnitudes.^50–53^ The TS-SCS method assumes a constant unscreened polarizability-to-〈*r*^3^〉 ratio and constant characteristic frequency (wp) for all charge states of a chemical element,^46^ but these assumptions are not realistic. Due to these assumptions, the TS and TS-SCS methods are inaccurate for systems with charged atoms.^54–57^ Bucko *et al.* showed the TS and TS-SCS methods severely overestimate polarizabilities for dense solids.^57^ The TS-SCS method has also not been optimized to work with conducting materials, and we show in Section 5.2 below the TS and TS-SCS methods sometimes predict erroneous polarizabilities even greater than for a perfect conductor. As discussed in Sections 4 and 5 below, the TS-SCS method overestimates directional alignment of fluctuating dipoles at large interatomic distances. We also show the TS-SCS method sometimes gives asymmetric AIM polarizability tensors and unphysical negative AIM polarizabilities.

Several research groups developed improvements to the TS-SCS method. Ambrosetti *et al.* introduced range separation to avoid double counting the long-range interactions in TS-SCS followed by MBD (aka MBD@rsSCS).^58^ MBD@rsSCS improves the accuracy of describing directional alignments of fluctuating dipoles at large interatomic distances.^58^ Bucko *et al.* used Iterative Hirshfeld (IH^51^) partitioning in place of Hirshfeld partitioning to compute 〈*r*^3^〉^AIM^.^55,57^ While this was an improvement, it did not address several problems mentioned above. For example, the TS-SCS/IH method still unrealistically assumed the unscreened polarizability-to-〈*r*^3^〉 ratio is the same for various charge states of a chemical element.^55,57^ This assumption was removed in the subsequent Fractionally Ionic (FI) method.^54^ However, the FI approach requires separate reference polarizabilities and C_6_ dispersion coefficients for all charge states of a chemical element.^54^ This is extremely problematic, because some anions that exist in condensed materials (*e.g.*, O^−2^) have unbound electrons in isolation.^59^ Although methods to compute charge-compensated reference ion densities have been developed,^53,55,59–64^ those methods do not presently extend to computing charge-compensated polarizabilities and C_6_ coefficients of ions. Thus, several problems with the TS-SCS approach have not been satisfactorily resolved in the prior literature.

In Gould *et al.*'s FI method, reference free atom polarizabilities were computed for various whole numbers of electrons and interpolated to find fractionally charged free atom reference polarizabilities.^54^ This yields different polarizability-to-〈*r*^3^〉 ratios for different charge states of the same chemical element.^54^ Due to the instability of highly charged anions, the −1 states of halogens were the only anions Gould *et al.* computed self-consistently.^54,65^ For other anions, Gould and Bucko resorted to using DFT orbitals from the neutral atoms to build non-self-consistent anions for polarizability and C_6_ calculations,^65^ but this severely underestimates the diffuseness of anions (*i.e.*, underestimates their polarizabilities and C_6_ coefficients). Unfortunately, this problem cannot be easily resolved by performing self-consistent calculations for all charged states, because some highly charged anions (*e.g.*, O^2−^)^59^ contain unbound electrons. This makes the FI method problematic for materials containing highly charged anions. Because FI was not included in VASP version 6b that we currently have access to, we did not perform FI calculations for comparison in this work.

In this article, we develop a new approach that resolves these issues. Our method uses DDEC6 (ref. 61 and 66–68) partitioning to provide accurate net atomic charges (NACs), atomic volumes, and radial moments as inputs. Our method has new scaling laws for the unscreened atomic polarizabilities, characteristic frequency (wp), and C_6_ dispersion coefficients. The different scaling behaviors of surface and buried atoms are included *via m*-scaling. Our approach accurately handles the variability in polarizability-to-〈*r*^3^〉 moment ratio for charged surface atoms while only requiring reference atomic polarizabilities, reference C_6_ coefficients, and reference radial moments for isolated neutral atoms. It uses a new self-consistent screening procedure to compute screened polarizability tensors and C_6_ coefficients. Our approach separates non-directional screening from directional screening of the dipole interaction tensor. This allows a conduction limit upper bound to be applied between non-directional and directional screening to ensure buried atoms do not have a screened polarizability above the conduction limit. Our method yields three different types of dipole polarizabilities: (a) induced static polarizabilities corresponding to a uniform applied external electric field, (b) isotropic screened polarizabilities suitable as input into polarizable force-fields, and (c) fluctuating polarizabilities that are used to compute C_6_ dispersion coefficients *via* the Casimir–Polder integral. When computing C_6_ dispersion coefficients, we use multi-body screening to taper off the dipole directional alignment at large interatomic distances. The self-consistent screening is applied incrementally. Richardson extrapolation provides high numeric precision. Through quantum Drude oscillator (QDO) parameterization, our method also yields higher-order polarizabilities (*e.g.*, quadrupolar, octupolar, *etc.*) and higher-order dispersion coefficients (*e.g.*, C_8_, C_9_, C_10_, *etc.*) for AIMs. Other important improvements include: improved damping radii, proportional partitioning of shared polarizability components to avoid negative AIM polarizabilities, iterative update of the spherical Gaussian dipole width, and AIM polarizabilities are symmetric tensors.

Our method was designed to satisfy the following criteria:

(1) The method should require only the system's electron and spin density distributions as input;

(2) The method should work for materials with 0, 1, 2, or 3 periodic boundary conditions;

(3) The method should give accurate results for charged atoms in materials while only requiring reference polarizabilities and reference C_6_ coefficients for neutral free atoms; (In this context, reference polarizabilities and reference C_6_ coefficients refer to those polarizabilities and C_6_ coefficients stored within the software program that are used during application of the method.)

(4) The method should give accurate results for diverse materials types: isolated atoms; small and large molecules; nanostructured materials; ionic, covalent, and metallic solids, *etc.*;

(5) The method should give accurate results for both surface and buried atoms;

(6) The method should yield both static polarizability tensors and polarizabilities suitable for constructing molecular mechanics force-fields;

(7) The method should accurately describe both short- and long-range ordering of dipole polarizabilities and C_6_ coefficients;

(8) The method should have less than approximately 10% average unsigned error on C_6_ coefficients and dipole polarizabilities for the benchmark sets studied here;

(9) The method should include estimates for higher-order AIM polarizabilities (*e.g.*, quadrupolar, octupolar, *etc.*) and dispersion coefficients (*e.g.*, C_8_, C_9_, C_10_, *etc.*);

(10) The method should have low computational cost for both small and large systems.

There are two main applications for this MCLF method. First, the polarizabilities and dispersion coefficients can be used to partially parameterize molecular mechanics force-fields. In addition to polarizabilities and C_6_ dispersion coefficients, those force-fields would also need to include net atomic charges (NACs), flexibility parameters (*e.g.*, bond, angle, and torsion terms), exchange–repulsion parameters, (optionally) charge penetration parameters, and optionally other parameters. Second, the dispersion coefficients can be used to partially parameterize DFT + dispersion methods.^23^ In addition to the C_6_ dispersion coefficients, an accurate DFT + dispersion method should also include higher-order dispersion (*e.g.*, C_8_, C_9_, and/or C_10_ terms) or multi-body dispersion (MBD) combined with an accurate damping function.^22,23^ (Partially analogous to the MBD@rsSCS method,^58^ range separation would be required to avoid double counting dispersion interactions when combining a MCLF variant with a MBD Hamiltonian.) Because DFT and molecular mechanics are widely used in computational chemistry, our new method can have widespread applications.

The remainder of this article is organized as follows. Section 2 contains the background information. Section 3 contains the new isolated atom scaling laws developed for MCLF. Section 4 describes the theory of the MCLF method. Section 5 contains calculation results of C_6_ coefficients and polarizabilities and comparisons to benchmark data. Section 6 is the conclusions.

## 2. Background information

### 2.1 Benchmarking methods

Experimental data and high-level quantum chemistry calculations were used as references in this work. Section 3.1 below describes reference polarizabilities and dispersion coefficients (C_6_, C_8_, and C_10_) for the isolated atoms. For diatomic molecules in Section 5.1, we computed static polarizability tensors using CCSD calculations combined with the “polar” keyword in Gaussian09 (ref. 69). As explained in Section 5.2 below, we set the reference static polarizability for dense solids to the lesser of the Clausius–Mossotti relation value and the conduction limit upper bound based on the experimental crystal structure geometry and dielectric constant.

For the small molecules in Section 5.3, reference static polarizabilities were obtained from published experiments. Experimental isotropic polarizabilities were extracted from dielectric constant or refractive index measurements having approximately 0.5% or less error.^70^ Refractive index can be measured by passing a light ray through a gas-phase sample.^71^ The polarizability *α*(*ν*) of the sample at frequency *ν* can then be calculated using3
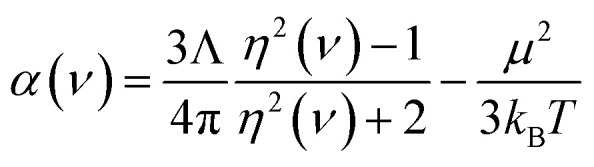
where *η* is the refractive index, *μ* is the dipole moment magnitude, *T* is absolute temperature, *Λ* is the volume per molecule, and *k*_B_ is Boltzmann's constant.^70^ (The last term in eqn (3) accounts for orientational polarizability.) Static or low-frequency dielectric constants *κ* were obtained by measuring the ratio of the capacitance of a set of electrodes with the sample material in-between to the capacitance of the same electrodes with vacuum in-between.^72^ The polarizability of a gas-phase sample can then be calculated using the Clausius–Mossotti relation:^73^4
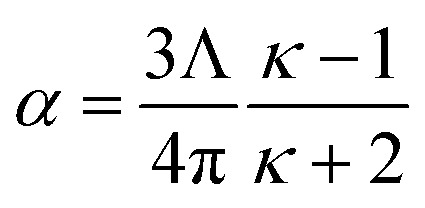


Reference C_6_ coefficients for the atom/molecule pairs (Section 5.4) were taken from the experimentally extracted dipole oscillator strength distribution (DOSD) data of Meath and co-workers^74,75^ as tabulated by Bucko *et al.*^55^

Time-dependent DFT (TD-DFT) and time-dependent Hartree–Fock (TD-HF) can be used to compute benchmark polarizabilities and dispersion coefficients. The Casimir–Polder integral is used to calculate C_6_ coefficients from polarizabilities at imaginary frequencies (imfreqs).^76^ For polyacenes (Section 5.5), reference C_6_ coefficients and isotropic static polarizabilities are from the TD-DFT calculations of Marques *et al.*^77^ For selected polyacenes, static polarizability tensor components were available as reference from Jiemchooroj *et al.*'s TD-DFT calculations.^78^ Jiemchooroj *et al.* found their TD-DFT results were similar to TD-HF, experimental (where available), and CCSD (where available) results. For fullerenes (Section 5.5), the reference C_6_ coefficients and isotropic static polarizabilities are from the TD-HF calculations of Kauczor *et al.*^116^ Kauczor *et al.* also obtained similar results using TD-DFT.^116^

### 2.2 Notation

A system may have either 0, 1, 2, or 3 periodic boundary conditions. In periodic materials, the term ‘image’ refers to a translated image of the reference unit cell. Each image is designated by translation integers (*L*_1_, *L*_2_, *L*_3_) that quantify the unit cell translation along the lattice vectors. The reference unit cell is the image designated by (*L*_1_, *L*_2_, *L*_3_) = (0, 0, 0). −∞ ≤ *L*_*i*_ ≤ ∞ along a periodic direction. *L*_*i*_ = 0 along a non-periodic direction. Similar to the notation previously used in the bond order article,^67^ a capital letter (A, B,…) designates an atom in the reference unit cell and a lowercase letter (a, b,…) designates an image atom. For example, *b* = (B, *L*_1_, *L*_2_, *L*_3_) denotes a translated image of atom B.

Let *R⃑*_B_ represent the nuclear position of atom B in the reference unit cell. Then, the nuclear position of a translated image is

5 *R⃑*_b_ = *R⃑*_B_ + *L*_1_*v⃑*^(1)^ + *L*_2_*v⃑*^(2)^ + *L*_3_*v⃑*^(3)^

where *v⃑*^(1)^, *v⃑*^(2)^, and *v⃑*^(3)^ are the lattice vectors. The distance between the nuclear position of atom A and the translated image of atom B is

6 *d*_Ab_ = *r*^AB,L^ = ‖*R⃑*_b_ − *R⃑*_A_‖

Cartesian components (*s* = *x*, *y*, *z*) of the vector from atom A's nuclear position to image b's nuclear position are represented by

7 *r*^AB,L^_s_ = *R⃑*_b_ − *R⃑*_A_

*r⃑*_A_ is the vector from the image of atom A's nuclear position to the spatial position *r⃑*:

8 *r⃑*_A_ = *r⃑* − *L*_1_*v⃑*^(1)^ − *L*_2_*v⃑*^(2)^ − *L*_3_*v⃑*^(3)^ − *R⃑*_A_

The length of this vector is represented by

9 *r*_A_ = ‖*r⃑*_A_‖

A stockholder partitioning method assigns a set of atomic electron densities {*ρ*_A_(*r⃑*_A_)} in proportion to atomic weighting factors {*w*_A_(*r*_A_)}

10 *ρ*_A_(*r⃑*_A_) = *ρ*(*r⃑*)*w*_A_(*r*_A_)/*W*(*r⃑*)

so that all sum to the total electron density11
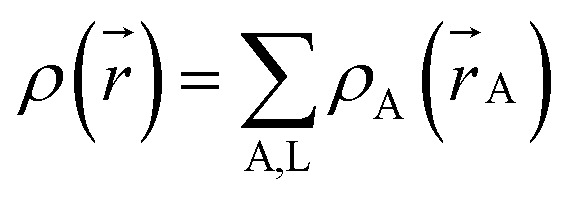
12
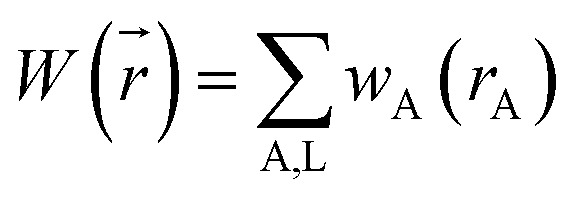
where summation over A, L means summation over all atoms in the material. The number of electrons *N*_A_ and net atomic charge (*q*_A_) assigned to atom A are

13 *N*_A_ = ∮*ρ*_A_(*r⃑*_A_)*d*^3^*r⃑*_A_ = *Θ*_A_ − *q*_A_

where *Θ*_A_ is the atomic number for atom A. As discussed in Section 2.4 below, different ways of defining {*w*_A_(*r*_A_)} produce different stockholder methods. The AIM radial moment of order *ϕ* is

14 〈(*r*_A_)^*ϕ*^〉 = ∮(*r*_A_)^*ϕ*^*ρ*_A_(*r⃑*_A_)*d*^3^*r⃑*_A_

〈*r*^2^〉, 〈*r*^3^〉, and 〈*r*^4^〉 are shorthand for 〈(*r*_A_)^2^〉, 〈(*r*_A_)^3^〉, and 〈(*r*_A_)^4^〉, respectively.

Since a particular dispersion-polarization model can be combined with different charge partitioning methods, we indicate the combination by the dispersion-polarization model followed by ‘/’ followed by the charge partitioning method. For example, TS-SCS/IH indicates the TS-SCS dispersion-polarization model using IH charge partitioning. Where our computed data are simply labeled ‘TS-SCS’, the DDEC6 charge partitioning method was used. Since all of the MCLF results reported in this paper used DDEC6 charge partitioning, we used the less precise but shorter term ‘MCLF’ in place of the full ‘MCLF/DDEC6’ designation. More generally, the MCLF dispersion-polarization model could potentially be combined with other charge partitioning methods (*e.g.*, MCLF/IH), but that is beyond the scope of the present study.

Calculating dispersion coefficients involves integrating polarizabilities over imfreqs. This is inconvenient in two respects. First, it is easier to deal with real-valued variables rather than imaginary-valued ones. Second, numeric integration from zero to infinite imaginary frequency is inconvenient, because infinity cannot be readily divided into finite intervals for numeric integration. Letting *ω* represent an imaginary frequency magnitude, we used the following variable transformation to solve these two problems:15
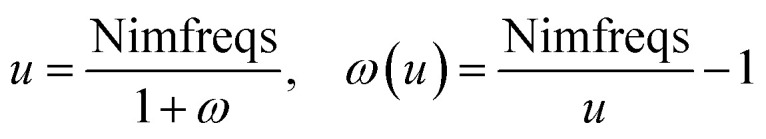
This conveniently transforms integration limits *ω* = [0, ∞) into *u* = [Nimfreqs, 0), which upon changing the integrand's sign gives integration limits *u* = (0, Nimfreqs]. As shown in the companion article, this allows convenient Rhomberg integration using integration points *u* = 1, 2,… Nimfreqs.^79^ In this article, *α*(*u*) denotes the polarizability at the imaginary frequency whose magnitude equals *ω*(*u*).

### 2.3 Details of the TS-SCS methodology

**Fig. 1:**
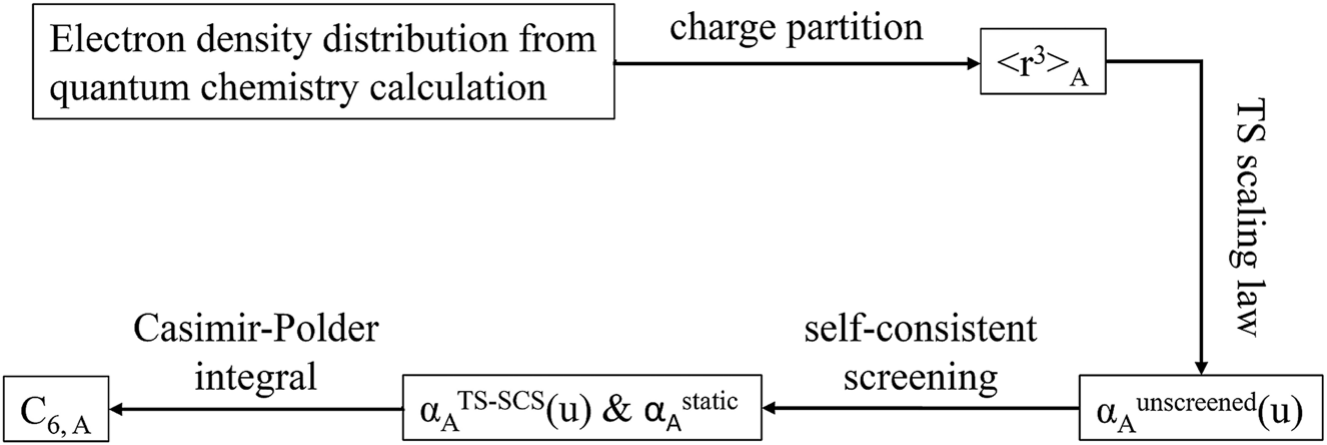
Flow diagram for TS-SCS method.

Fig. 1 is a flow diagram of the TS-SCS method. Bucko *et al.*^56^ gave the step-by-step calculation of the self-consistent screening process. Here, we follow the presentation of Bucko *et al.*, except using the variable substitution of eqn (15). For atoms A and B, they define a many-body polarizability matrix *P*, with its inverse *Q* having the form16
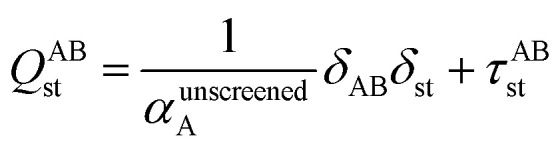
where s, t designate Cartesian components. Square matrices *P* and *Q* have *x*, *y*, and *z* spatial indices for every atom to give a total of 3Natoms rows. The last term on the right-hand side is the dipole interaction tensor which has the form17
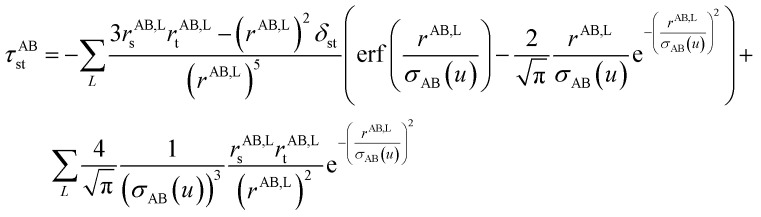
where summation over *L* means summation over all periodic translation images (if any). *σ*_AB_(*u*) is the attenuation length for the pair of atoms A and B18
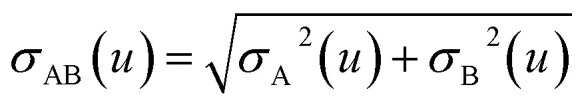


The spherical Gaussian dipole width is obtained from19
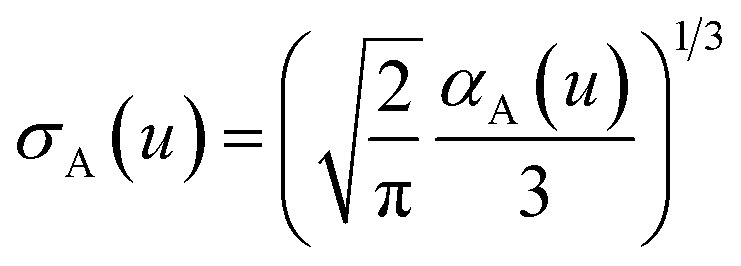
and the isotropic dynamical atomic polarizability is20
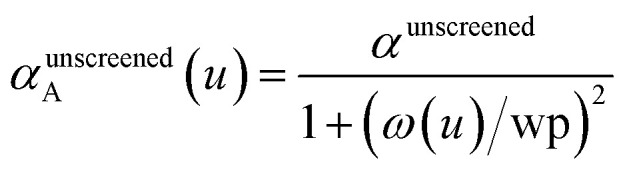


In the TS and TS-SCS methods,

21 *α*^unscreened^ = *α*^TS^

where *α*^TS^ is calculated by eqn (2). AIM polarizability tensors are computed using the partial contraction22
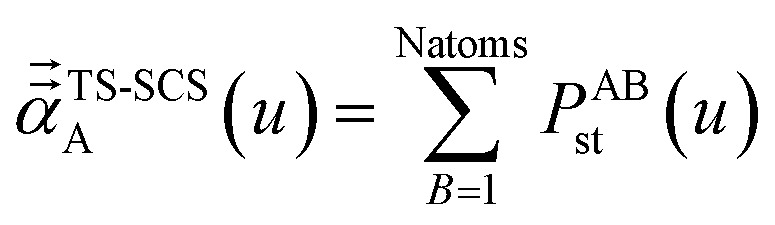
with the static polarizability tensor corresponding to *u* = Nimfreqs. The screened frequency-dependent isotropic polarizability is computed as one third of the trace of the three-by-three polarizability tensor obtained by partial contraction of *P*23
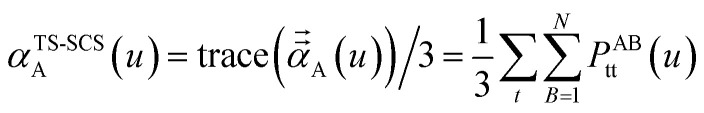


These are fed into the Casimir–Polder integral expressed in terms of *u* (see companion article for derivation^79^)24
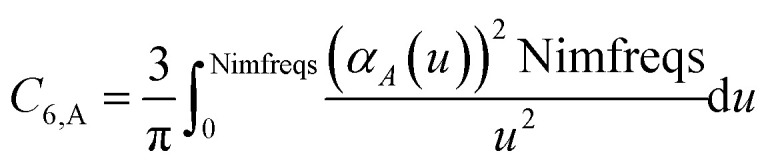
to compute C_6,A_.

### 2.4 Electron density partitioning methods

In Hirshfeld partitioning introduced in 1977, atoms are partitioned to resemble the neutral reference atom.^45^ This makes the atoms tend to have lower charge than they should have.^50^ The iterative Hirshfeld partition (IH) keeps updating the reference with the charge state of the atom.^51^ However, this approach leads to the runaway charge problem in some cases.^61^ As shown in ref. 57 and Section 5.2 below, using TS or TS-SCS with Hirshfeld or IH partitioning overestimates polarizabilities for dense solids.

Manz and Sholl presented DDEC1 and 2 atomic population analysis methods in 2010.^60^ By simultaneously optimizing the AIM density distributions to be close to spherically symmetric and to resemble charge-compensated reference ion densities, this method can give chemically meaningful NACs and accurate electrostatic potential for some materials, but was later found to give runaway charges in other materials. In 2012, Manz and Sholl presented the DDEC3 method that partially fixes the runaway charge problem by increasing the optimization landscape curvature *via* conditioned reference densities and imposing an exponential decay constraint on each atom's electron density tail.^53^

Manz and Limas presented DDEC6 partitioning in 2016.^61,66^ This method fixes the runaway charge problem. Also, new constraints are added to the decay rate of the buried atom tails. The weighted spherical average improves the effect of spherical averaging during charge partitioning. Along with guaranteed convergence in seven steps, this method is very accurate, cost efficient, and produces chemically meaningful NACs.^61,66,68^ In 2017, Manz published a new method for computing bond orders, which is based on DDEC6.^67^

## 3. New isolated atom scaling laws

### 3.1 Reference data

The reference polarizabilities (*α*_CCSD_) used in this work are our calculated polarizabilities using the CCSD method with def2QZVPPDD basis set (the def2QZVPPDD basis set is defined in the ESI†). We tested two different methods: (a) using Gaussian09 (ref. 69) keyword ‘polar’ to compute the molecular static polarizability tensor using perturbation response theory and (b) using Gaussian09 keyword ‘field’ to manually apply a small constant external electric field *E⃑* in order to calculate the molecular static polarizability tensor as 
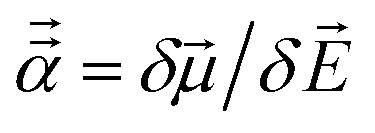
 where *μ→* is the molecular dipole moment. However, many of the manual (*i.e.*, keyword = ‘field’) calculations did not converge and the converged results were not as consistent with Gould and Bucko's data^65^ as the perturbation response calculations. So we decided to use the perturbation response calculations (*i.e.*, keyword = ‘polar’) for all elements except Y. For Y, the keyword = ‘field’ polarizability was used, because the perturbation response calculation gave an unreasonably low polarizability of 88.98 compared to *α*_Gould_ = 163 while the manually applied field polarizability of 158.81 was close to Gould and Bucko's value and followed the trend of neighboring elements: *α*_Sr,CCSD_ = 204.51 *α*_Zr,CCSD_ = 143.47.

**Fig. 2:**
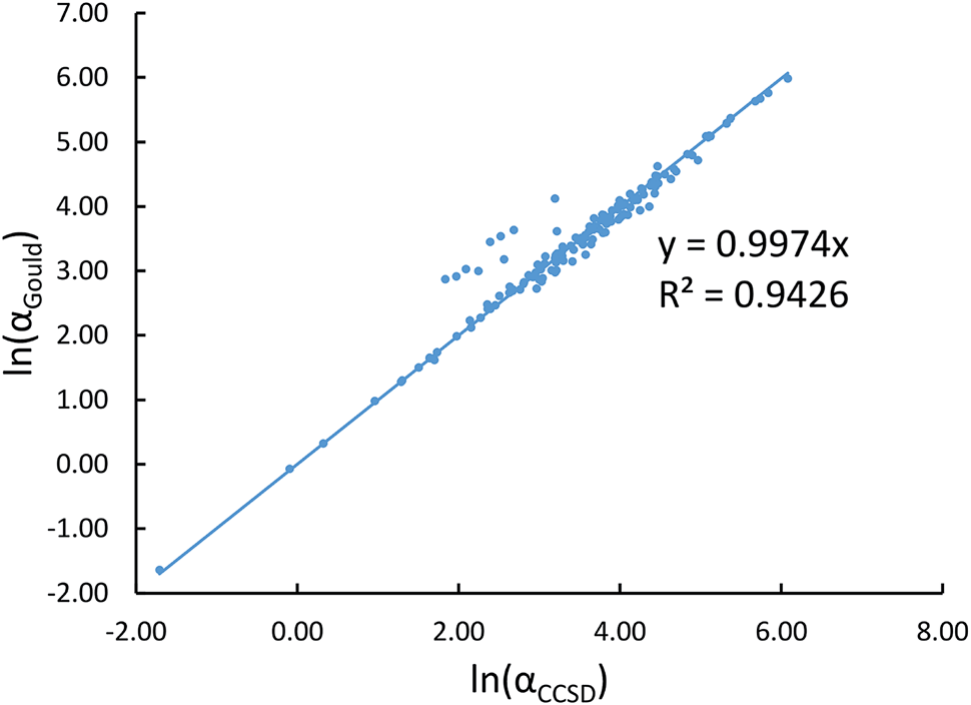
Plot of our CCSD isolated atom reference polarizabilities *versus* Gould and Bucko's values.

Fig. 2 shows that our calculated polarizabilities are mostly consistent with Gould and Bucko's values. We used *α*_CCSD_ rather than *α*_Gould_ as the reference free atom polarizability, because our radial moments come from the same CCSD calculations as used to compute *α*_CCSD_. For elements using a relativistic effective core potential (RECP) in the def2-QZVPPDD basis set, the radial moments of core electrons replaced by the RECP are added back in using a reference core density library; thus yielding effective all-electron radial moments. Since Gould and Bucko used the aufbau principle for electron configurations of transition metal atoms, their calculations do not necessarily correspond to the ground state spin multiplicity for transition metal atoms.^65^ Recently, Schwerdtfeger and Nagle published a set of recommended polarizabilities for chemical elements 1 to 120 (except livermorium), but those were not used in our study because they do not have a corresponding set of C_6_ values.^80^

CCSD in Gaussian09 does not have the capability of calculating C_6_ coefficients. Therefore, to maximize consistency between the free atom reference radial moments, polarizabilities, and C_6_ coefficients, our reference C_6_ coefficients were calculated as25
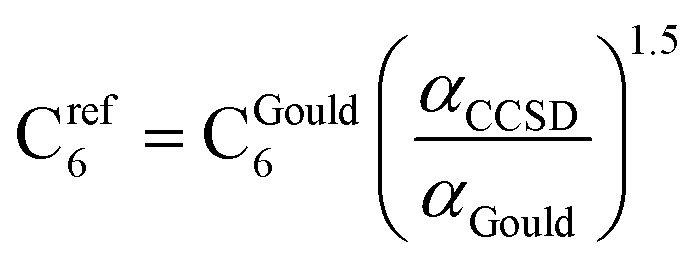
where *α*_Gould_ and C^Gould^_6_ are Gould and Bucko's values using TD-DFT.^65^ This C_6_ rescaling makes C^ref^_6_ correspond to *α*_CCSD_, which corresponds to the computed radial moments. The 3/2 power occurs in eqn (25), because *α* and C_6_ for a free atom are approximately proportional to the free atom's effective radius to the fourth and sixth powers, respectively (see Table S1 of ESI†).

The reference *α*, C_6_, wp, *r* moments, and damping radii are listed in the ESI.† This dataset contains neutral elements 1–86 except the f-block elements (58–71). The reason for excluding the f-block and heavier elements is the def2QZVPPDD basis set we are using does not include these elements. The dataset also includes +1 cations of elements 3–7, 11–17, 19–57 and 72–86 and −1 anions of F, Cl, Br, I, and At. These ions were also self-consistently calculated by Gould and Bucko.^65^ Gould and Bucko included additional anions which were not computed self-consistently, and we omit these because self-consistent polarizabilities are unavailable.^65^ The reference wp were calculated from the CCSD polarizability and the C^ref^_6_ using^81^26
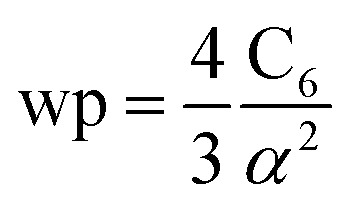


The reference C_8_ and C_10_ are from several sources compiled by Porsev and Derevianko^82^ and Tao *et al.*^83^ and are listed in the ESI.† This dataset contains H, Li, Na, K, Rb, Cs, He, Ne, Ar, Kr, Xe, Be, Mg and Ca. Most of these reference values are based on correlated quantum chemistry calculations.

### 3.2 Deriving the new scaling laws

**Fig. 3:**
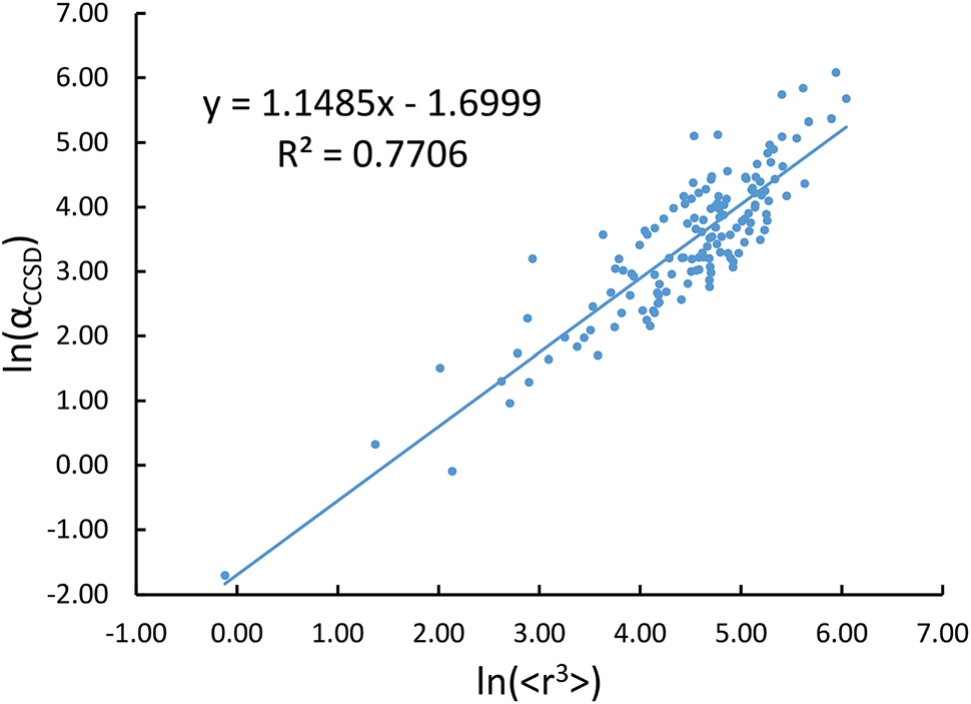
Plot showing ln(*α*_CCSD_) *versus* ln(〈*r*^3^〉) exhibits a weak correlation (*R*^2^ = 0.7706).

Johnson and Becke assumed that for a given chemical element, the polarizability of atoms in a material should be proportional to the 〈*r*^3^〉 moment of the atom-in-material.^42^ This assumption was subsequently adopted by Tkatchenko and Scheffler when formulating the TS method.^12^ Of course, this is not the same as assuming polarizabilities of isolated atoms across different chemical elements should be proportional to their 〈*r*^3^〉 moments. As pointed out by Gould, the isolated atom polarizabilities are not proportional to their 〈*r*^3^〉 moments.^49^Fig. 3 is a plot of ln(polarizability) *versus* ln(〈*r*^3^〉) for the isolated atoms. This plot shows a weak correlation (*R*^2^ = 0.7706). This motivated us to develop a new polarizability scaling law that applies both to isolated atoms and to atoms-in-materials across different chemical elements.

We tested seven models containing electron number and different combinations of the *r* moments as the independent variables and *α*, C_6_, and wp as the dependent functions. Table 1 lists the *R*^2^ values of the 7 models. The coefficients were obtained by least squares fitting of a linear combination of the log values of *r* moments and electron numbers to *α*, C_6_, or wp using a Matlab program we wrote. For example, the entry in row 2 and column 2 in Table 1 is the *R*^2^ value of 0.6626 obtained by fitting log(〈*r*〉) and log of electron number to log(*α*_CCSD_). The results show that using only one *r* moment does not yield high *R*^2^ value. Combinations of two or more *r* moments give higher *R*^2^ values, with the 〈*r*^3^〉 & 〈*r*^4^〉 model giving the best average performance.

**Table:** *R* ^2^ values for fitted parameters using CCSD *r* moments for isolated atoms

Model	*α*_CCSD_	C_6_	wp
N & 〈*r*〉	0.6626	0.7619	0.3922
N & 〈*r*^2^〉	0.8021	0.8809	0.5489
N & 〈*r*^3^〉	0.8831	0.9414	0.6615
N & 〈*r*^4^〉	0.9242	0.9672	0.7317
N, 〈*r*^2^〉 & 〈*r*^3^〉	0.9457	0.973	0.8222
N, 〈*r*^2^〉 & 〈*r*^4^〉	0.9545	0.9772	0.8452
N, 〈*r*^3^〉 & 〈*r*^4^〉	0.9549	0.977	0.8494

Table 2 lists parameters for the 〈*r*^3^〉 & 〈*r*^4^〉 model. The proposed relations between *α*, wp, and C_6_ and the parameters have the following form, where all quantities are expressed in atomic units:27
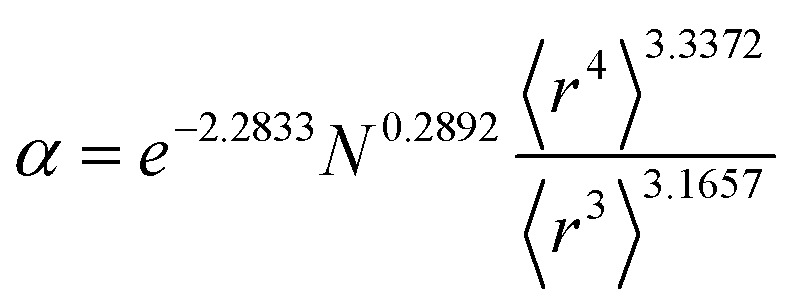
28
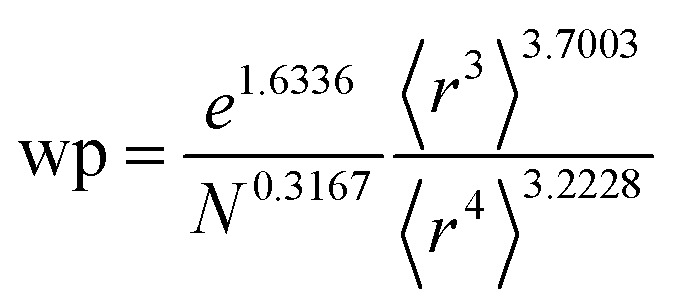
29
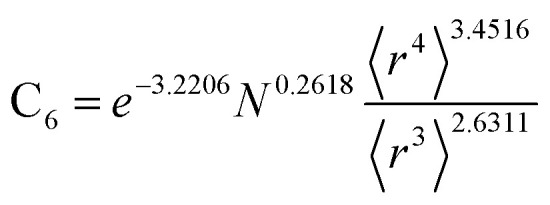


**Table:** Parameter coefficients for the new scaling law

*α*		C_6_		wp	
Component	Coefficient	Component	Coefficient	Component	Coefficient
Constant	−2.2833	Constant	−3.2206	Constant	1.6336
N	0.2892	N	0.2618	N	−0.3167
〈*r*^3^〉	−3.1657	〈*r*^3^〉	−2.6311	〈*r*^3^〉	3.7003
〈*r*^4^〉	3.3372	〈*r*^4^〉	3.4516	〈*r*^4^〉	−3.2228

**Fig. 4:**
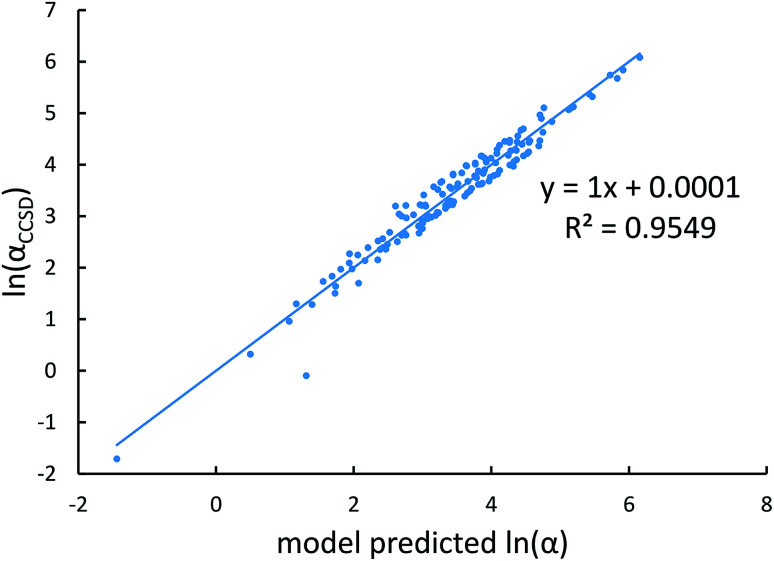
Model predicted ln(*α*) *versus* reference ln(*α*).

**Fig. 5:**
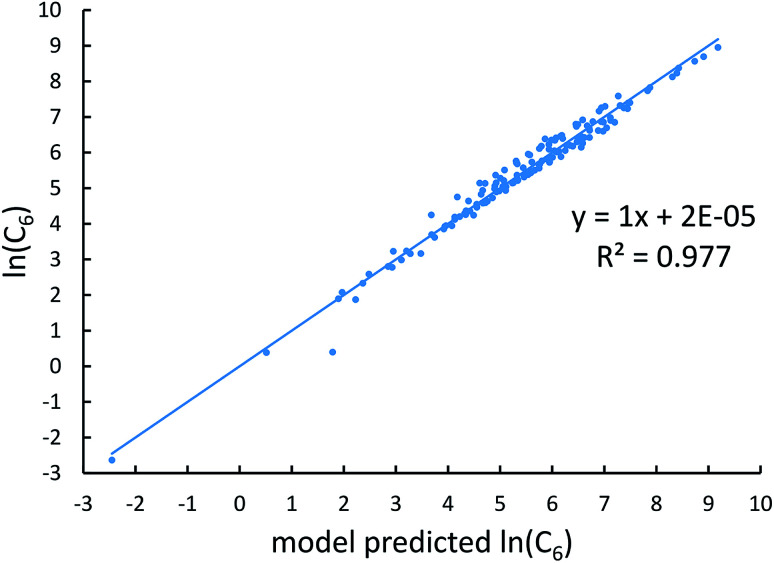
Model predicted ln(C_6_) *versus* reference ln(C_6_).

Fig. 4, 5, and 6 show strong correlation between the model predicted *α*, C_6_, and wp and the reference data with *R*^2^ values of 0.9549, 0.977, and 0.8494, respectively.

**Fig. 6:**
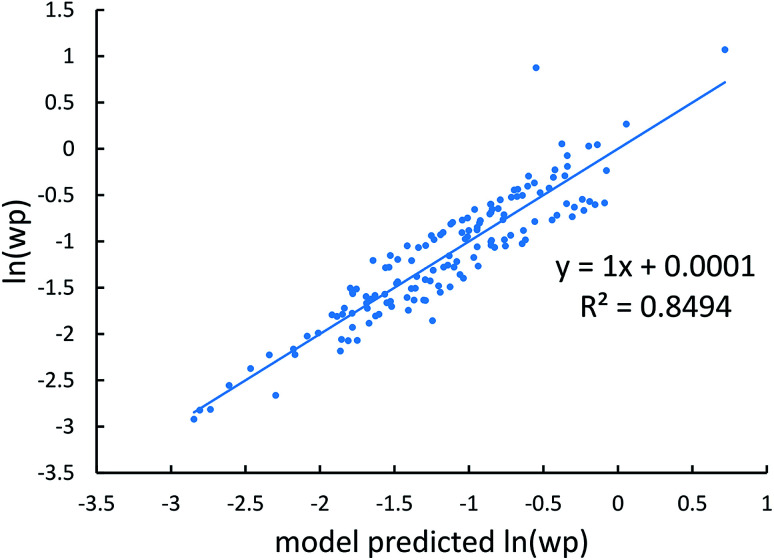
Model predicted ln(wp) *versus* reference ln(wp).

To test the robustness and transferability of the different models, the following tests were performed as shown in Table 3. The “PW91 refitted” column are the *R*^2^ values obtained by refitting the model parameters with *r* moments from PW91. Manz and Limas performed these PW91 calculations in Gaussian09 using an all-electron fourth-order Douglas–Kroll–Hess relativistic Hamiltonian with spin–orbit coupling (DKHSO) and the MUGBS basis set near the complete basis set limit employing a finite-size Gaussian nuclear model.^60,61^ The “PW91 predicted” column are the *R*^2^ values calculated using CCSD model parameters from Table 1 but with PW91 *r* moments instead of CCSD *r* moments. Table 3 shows that the 〈*r*^3^〉 & 〈*r*^4^〉 model has the highest *R*^2^ in both tests; therefore, this model is the most robust and transferable.

**Table:** *R* ^2^ values for parameters using PW91 *r* moments

Model	PW91 refitted			PW91 predicted		
	*α*_CCSD_	C_6_	wp	*α*_CCSD_	C_6_	wp
N, 〈*r*^2^〉 & 〈*r*^3^〉	0.8785	0.9130	0.7571	0.8127	0.8658	0.6181
N, 〈*r*^2^〉 & 〈*r*^4^〉	0.8904	0.9176	0.7942	0.8276	0.8706	0.6780
N, 〈*r*^3^〉 & 〈*r*^4^〉	0.8942	0.9181	0.8159	0.8345	0.8726	0.7104

Hence, the new polarizability scaling law for an isolated atom is30
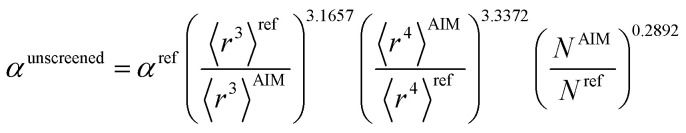
where *N* is the number of electrons, the superscript “ref” means the value of the neutral atom reference, and “AIM” means the value for atom-in-material after partitioning. The new wp scaling law for an isolated atom is31
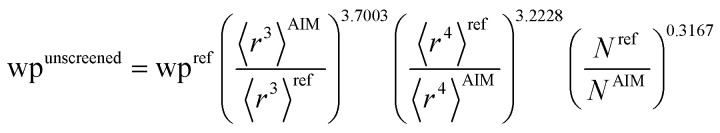
C^unscreened^_6_ for an isolated surface atom is then computed *via*eqn (26). These scaling laws allow different charge states of an atom to be accurately described while using only reference polarizability and wp values for a neutral free atom of the same element. For *α*, the effective power of the effective radius is 4 × 3.3372 − 3 × 3.1657 = 3.8517, which is approximately 4. For wp, the effective power of the effective radius is 3 × 3.7003 − 4 × 3.2228 = −1.7903, which is approximately −2. Scaling laws for non-isolated atoms will be addressed in Section 4 below.

### 3.3 Higher-order dispersion coefficients and quantum Drude oscillator parameters

In this section, we consider higher-order dispersion coefficients C_8_, C_9_, and C_10_ and their mixing rules. The contribution of the three-body C_9_ term to the dispersion energy is typically less than 10%.^11^ Nevertheless, McDaniel and Schmidt^84^ showed that in order to obtain accurate results from force-field simulations for condensed phases, the three-body term (*E*^ABC^) should be included. Tang and Toennies showed that the attractive potential at well depth for two free atoms is mainly composed of C_6_, C_8_, and C_10_ with contributions of roughly 65%, 25%, and 7% respectively.^85^ The rest comes from higher-order terms. Because the C_8_, C_9_, and C_10_ terms have modest contributions, we decided to include them in our model.

The C_8,A_ coefficient describes the fluctuating-dipole-fluctuating-quadrupole two-body dispersion interaction between atoms of the same type. We defined two groups (with all quantities expressed in atomic units) for least-squares fitting to obtain a model for C_8,A_:32
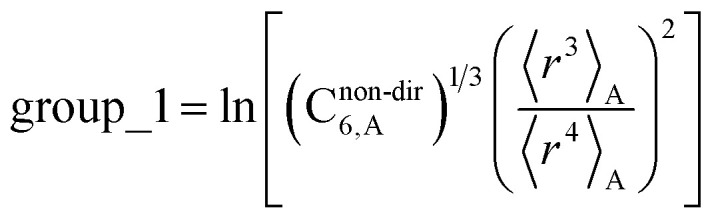
33
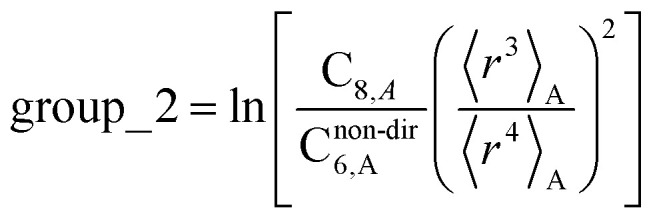


**Fig. 7:**
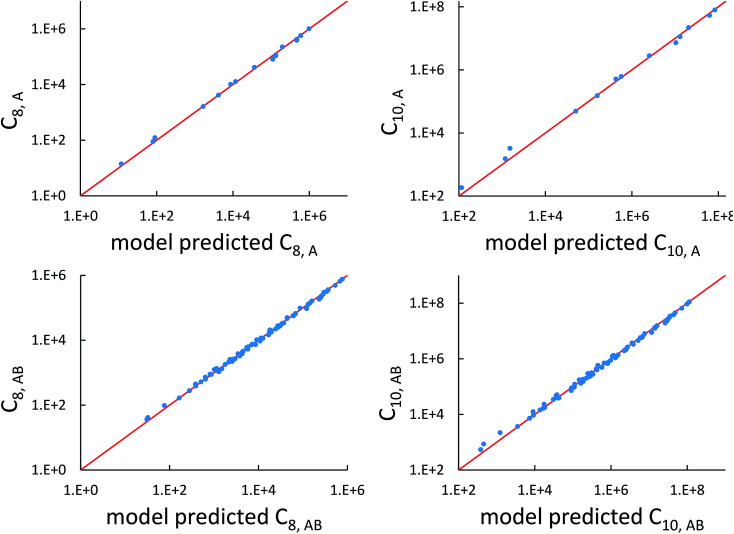
Model predicted C_8,A_, C_10,A_, C_8,AB_, and C_10,AB_*versus* reference values.

The reason for using 〈*r*^3^〉 and 〈*r*^4^〉 is that these are the same *r* moments used in models discussed above. Since C_8,A_ describes the fluctuating dipole–quadrupole coupling while C_6,A_ describes the fluctuating dipole–dipole coupling, there is no reason to believe directional effects on C_8,A_ follow those on C_6,A_. Therefore, our correlations for higher-order dispersion coefficients (*i.e.*, C_8_, C_9_, and C_10_) do not include directional coupling. C^non-dir^_6,A_ is obtained using the imfreq-dependent non-directionally screened atomic polarizability *α*^non-dir^_A_(*u*) in the Casimir–Polder integral. Linear fitting was performed to obtain the slope and intercept for group 2 as a function of group 1. The results were 0.8305 for the slope and 1.7327 for the intercept yielding:34
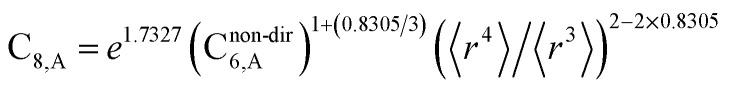
The top left panel of Fig. 7 shows strong correlation between the model predicted C_8,A_ and the reference data^82,83^ for selected isolated atoms with MARE of 14.6%.

The Quantum Drude Oscillator (QDO) model provides a natural framework for describing multibody polarizability and dispersion interactions beyond the dipole approximation, including quadrupolar, octupolar, and high-order interactions.^9,86,87^ A QDO consists of a negative pseudoparticle coupled *via* a harmonic potential to a pseudonucleus.^9,86,87^ This harmonic coupling produces a Gaussian charge distribution.^9,86^ In our model, one QDO is centered on each atom in the material. Each QDO is completely described by three parameters: (a) an effective mass (*m*^QDO^), (b) an effective charge (*q*^QDO^), and (c) an effective frequency (wp^QDO^).^9,86^ Using literature relations^9^ applied to our non-directionally screened quantities yields35
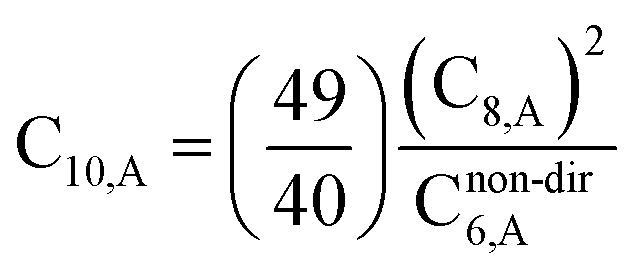
36
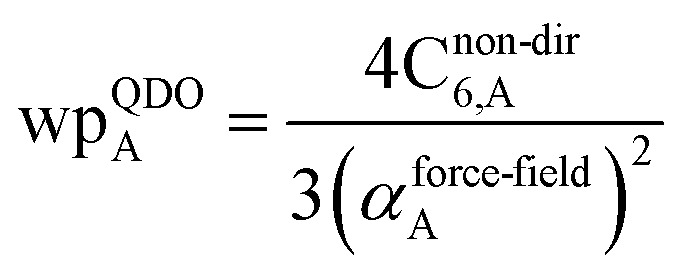
37
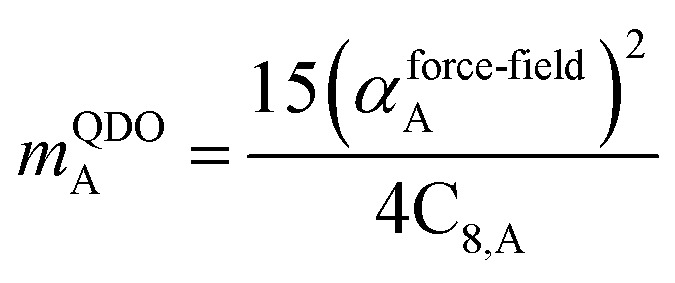
38
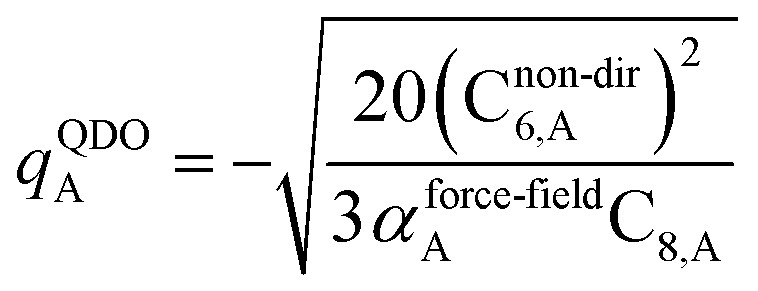
39
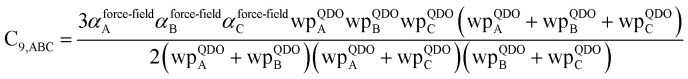
40
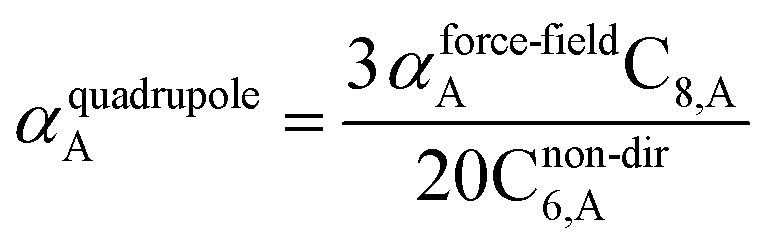
41
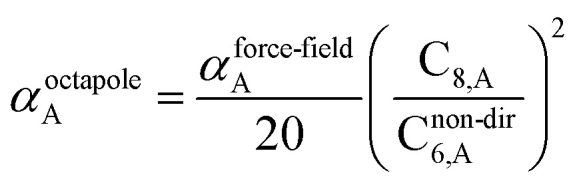
42
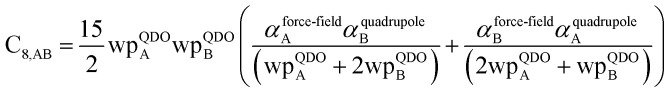
43
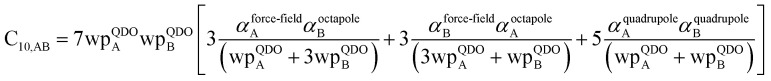
The C_9,ABC_ QDO mixing rule^9^ is similar to that described much earlier by Tang using a Padé approximation.^81^ The three-body fluctuating dipole–dipole–dipole interaction energy term is *E*_9_ = C_9,ABC_(1 + 3 cos(∠*a*)cos(∠*b*)cos(∠*c*))/(*R*_AB_*R*_AC_*R*_BC_)^3^ where ∠*a*, ∠*b*, and ∠*c* are the angles of the ABC triangle.^81,164–166^ The top right panel of Fig. 7 shows strong correlation between the model predicted C_10,A_ and the reference data^82,83^ for selected isolated atoms with MARE of 18.6%.

When constructing a force-field using the MCLF dispersion coefficients, care should be taken not to double-count the three-body dipole–dipole–dipole interactions. Specifically, the MCLF directional screening (*i.e.*, C^screened^_6_) already includes the three-body dipole–dipole–dipole interactions for the calculated system. (These were included *via* the directional dipole interaction tensor which was used in turn to compute C^screened^_6_.) For example, if C^screened^_6_ is computed using the MCLF method for a single benzene molecule, then the intramolecular dipole–dipole–dipole interactions are already included *via* the C^screened^_6_ coefficient term, but the intermolecular dipole–dipole–dipole interactions are not already included and should be added in the force-field using the C_9_ coefficient term. In this case, the force-field's C_9_ three-body term should be constructed to include atom triplets from two or three molecules, but not from a single molecule.

Fig. 7 compares model predicted to reference C_8_ and C_10_ dispersion coefficients. Reference values are from Porsev and Derevianko^82^ and Tao *et al.*^83^ and sources cited therein. Chemical elements included in Fig. 7 are: (a) the alkali metals Li, Na, K, Rb, Cs, (b) the noble gases He, Ne, Ar, Kr, Xe, (c) hydrogen H, and (d) the alkaline earths Be, Mg, Ca. Plotted C_8,AB_ and C_10,AB_ included 73 different heteroatomic pairs (see ESI† for details) formed from a subset of these elements.

### 3.4 Extension to element 109

The CCSD reference data are missing for elements 58–71 and 87–109 because Def2QZVPPDD basis set is not available for those elements. In order for the method to be more applicable, we used eqn (26)–(28) to estimate the reference polarizability, wp, and C_6_ for these elements. This is a reasonable approach since Table 3 already showed that CCSD model with PW91 *r* moments yields reasonable results. For these elements, the PW91 〈*r*^3^〉 and 〈*r*^4^〉 moments required as inputs were taken from all-electron fourth-order DKHSO calculations with MUGBS basis set near the complete basis set limit employing a finite-size Gaussian nuclear model.^60,61^ Furthermore, data for La (element 57, an f block element) is available for both CCSD and PW91. Comparing its PW91 *α* and wp with CCSD ones, the change is 5.4% and 2.7% respectively. The reference data for elements 58–71 and 87–109 are listed in ESI.†

## 4. Theory of MCLF method

### 4.1 New polarizability component partition

During tests, we noticed TS-SCS sometimes gives negative atomic polarizabilities. For example, in the ZrO molecule, the polarizability of oxygen is −0.607 from TS-SCS. This causes subsequent methods depending on the TS-SCS method to fail: (a) the C_6_ coefficient from the Casimir–Polder integral will be unphysical, (b) corresponding vdW radii in the TS-SCS method will be complex (since they depend on the cubed root of the polarizability),^12^ and (c) MBD, MBD@rsSCS, and related methods require non-negative AIM polarizabilities as input.^46,54,58^ In TS-SCS, the partial contraction of P follows the assumed form of Applequist *et al.*:^31^44
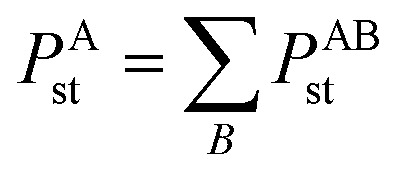
This sometimes yields negative AIM polarizabilities, because the mixed contribution *P*^AB^_st_ (which might be negative) between an atom A with small polarizability and an atom B with large polarizability can surpass the magnitude of *P*^AA^_st_.

In our new method, atoms with larger pre-screened polarizability get a proportionally larger piece of the screening mixed polarizability contribution:45
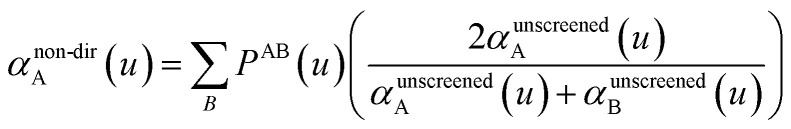
46
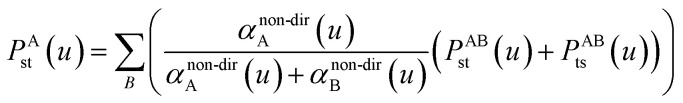
47
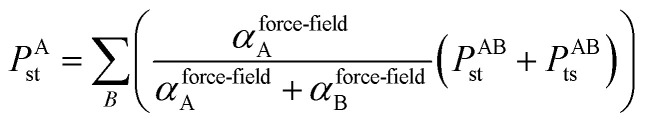
Eqn (45)–(47) correspond to the non-directional, fluctuating, and static polarizabilities, respectively. After this new partition is applied, the oxygen in ZrO has the MCLF polarizability of 6.754. Also, eqn (46) ensures the AIM polarizability *P*^A^_st_ is a symmetric tensor just like the total polarizability tensors^88,89^ of all molecules. In contrast, the TS-SCS polarizability tensor for an atom-in-material is sometimes asymmetric with respect to the spatial coordinates (*e.g.*, the *xy* and *yx* components are different). As an example, the ESI† contains the TS-SCS and MCLF results files for dibromomethane, where the TS-SCS *yz* and *zy* polarizability components for the last Br atom were 4.05 and 7.31, respectively; the MCLF method gave 5.74 for both components.

**Fig. 8:**
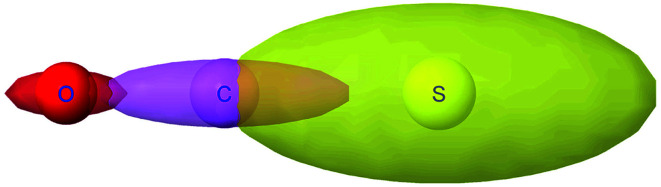
Illustration of MCLF atom-in-material polarizability tensors for the carbonyl sulfide molecule. The sulfur atom had much higher polarizability than the carbon and oxygen atoms. All three atoms showed enhanced polarizability along the bond direction. Only the relative sizes and shapes of the ellipsoids were drawn to scale.

The symmetric AIM polarizability tensor can be visualized by plotting the ellipsoid^88,89^48
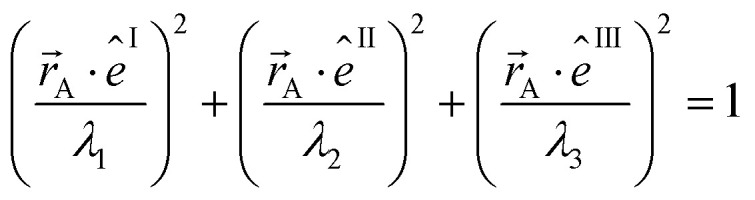
where *λ*_1_, *λ*_2_, and *λ*_3_ are its three eigenvalues and *ê*^I^, *ê*^II^, and *ê*^III^ are the corresponding mutually orthogonal eigenvectors. Fig. 8 plots AIM polarizability tensors for the carbonyl sulfide molecule. All three atoms showed enhanced polarizability along the bond direction. The atom-in-material polarizability along a unit direction *k̂* is quantified by the projection 
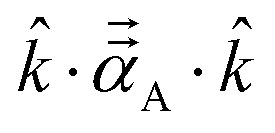
. Choosing *k̂* parallel to the inter-nuclear direction gives the bond polarizability of two bonded atoms: 
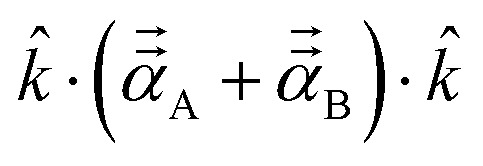
.^88,89^ Of interest, Raman spectrum peak intensities are proportional to the change in projected polarizability as vibration occurs.^90–92^

### 4.2 Polarizability upper bound

Consider a perfectly conducting plate with thickness *d* in an external electric field *E*_ext_ applied perpendicular to its surface. From Gauss' Law, the two faces perpendicular to *E*_ext_ develop surface charge densities *σ*′ = ±*ε*_0_*E*_ext_ and form a dipole moment *μ* = *σ*′*d* × area = *ε*_0_*E_ext_d* × area. The local polarizability must be computed based on the electric field (*E*_loc_) felt by each local volume element within the material. In this geometry, *E*_loc_ = *E*_ext_/2 is the average of the field before (*E*_ext_) and after (*E*_final_ = 0 inside the conductor) polarization. Its local polarizability is *p* = *μ*/*E*_loc_ = 2*ε*_0_*d* × area, which in atomic units (4π*ε*_0_ = 1 in atomic units) gives *α* = *p*/(4π*ε*_0_) = *d* × area/(2π), also referred to as the polarizability volume since it carries volume units. Thus, the local polarizability-to-volume ratio of the perfectly conducting plate is 1/(2π) in atomic units. For this geometry, the overall polarizability-to-volume ratio (*p* = *μ*/*E*_ext_) is 1/(4π).

As a second example, consider a sphere of radius *R* and dielectric constant *κ* placed in a constant externally applied electric field *E*_ext_ along the *z*-direction. This sphere will develop a dipole moment of^93^49
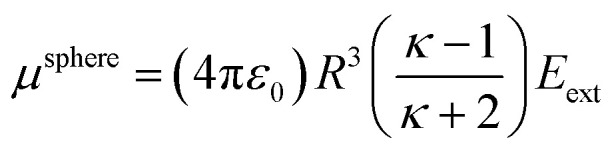
Therefore, its overall polarizability in atomic units is *R*^3^(*κ* − 1)/(*κ* + 2), and its overall polarizability-to-volume ratio is (3/(4π))((*κ* − 1)/(*κ* + 2)) in atomic units. The solution for a perfect conductor can be obtained by taking the limit *κ* → ∞ which yields 3/(4π) as the overall polarizability-to-volume ratio for the conducting sphere.

In theory, the polarizability caused by distortion of the electron cloud for fixed nuclear positions should be less than or equal to that of a perfect conductor. Comparing the above results for the conducting plate and conducting sphere shows the overall polarizability-to-volume ratio of a perfect conductor is shape-dependent; this is due to directional interactions within the material. To address this issue, we apply a conduction limit upper bound during non-directional screening, before any directional screening occurs. Applying the conduction limit upper bound before directional screening allows it to be based on the local polarizablity-to-volume ratio instead of the overall polarizability-to-volume ratio. The local polarizability-to-volume ratio should be approximately independent of the material's shape. Therefore, the local polarizability-to-volume ratio of the conducting plate is operational for the non-directional screening. So we defined the polarizability upper bound of atom A as50
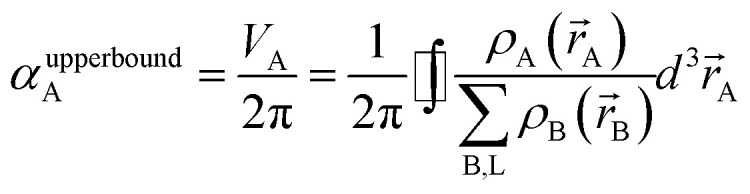
where *V*_A_ is the volume of atom A in the material.

During MCLF calculation, if the calculated non-directionally screened polarizability is higher than this upper bound, the result will be replaced by the upper bound polarizability. This procedure is carried out by a smooth minimum function

51 *α*_A_ = smooth_min(*α*^est^_A_, *α*^upperbound^_A_)

52
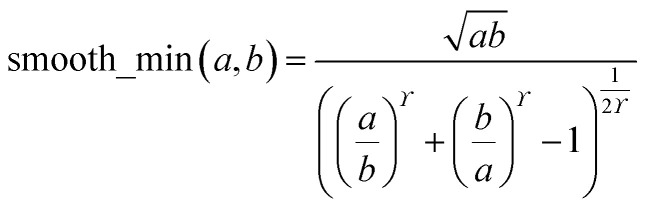
where *ϒ* = 25 because this value provides a smooth curve with less than 0.58% deviation from max(min(*a*, *b*), 0). If either *a* or *b* is ≤0, the function is set to return a value of 0. Using a smooth curve, instead of simply min(*a*, *b*), is required to ensure the forces (*i.e.*, energy derivatives with respect to atom displacements) are continuous functions.

### 4.3 M scaling to describe both surface and buried atoms

Section 3.2 above shows the polarizability of an isolated atom scales approximately proportional to its effective radius to the 4th power. Brinck *et al.* showed the polarizability of a polyatomic molecule is approximately proportional to its volume divided by an effective electron removal energy.^94^ As shown in Section 4.2, the polarizability of a conducting plate or sphere is proportional to its volume. For non-conducting fluids, the Clausius–Mossotti relationship describes a polarizability-to-volume ratio that is a weak function of the dielectric constant.^73^ This implies the polarizability-to-volume ratio of a buried atom is approximately proportional to its volume. This means the atom-in-material polarizability transitions from approximately 4th power to 3rd power dependence on the atom's effective radius as the atom goes from isolated to buried.

We define *m* to quantify how exposed an atom is:53
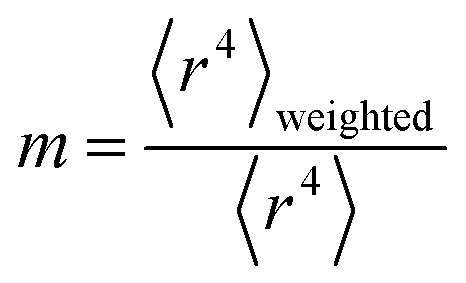
54
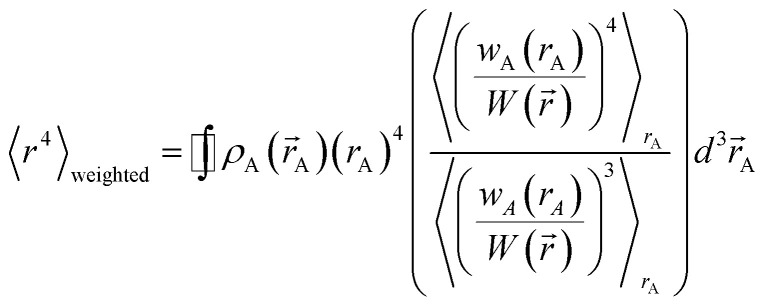
where *ρ*_A_(*r⃑*_A_) is the electron density of atom A, *w*_A_(*r*_A_)/*W*(*r⃑*) is the fraction of the total electron density *ρ*(*r⃑*) at position *r⃑* that is assigned to atom A, and 〈 〉_r_A__ means the spherical average. *m* equals 1 for an isolated atom and goes towards zero when an atom gets more and more buried.

The modified unscreened scaling law for *α* now has the form55
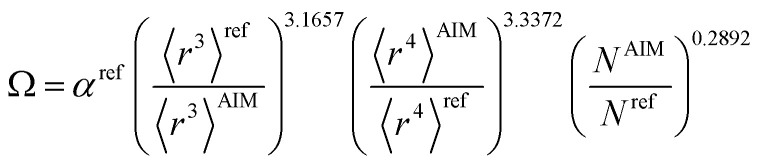

56 *α*^unscreened^ = (*C*〈*r*^3^〉)^(1−*m*)^Ω^*m*^

where *C* is a constant to be determined. When *m* = 1, it becomes the isolated atom scaling law in Section 3. When *m* = 0, it becomes57
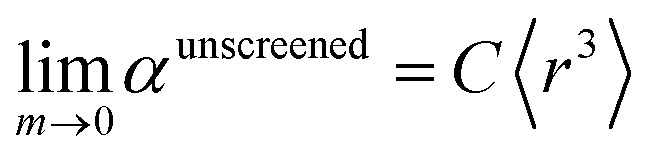


There are two primary justifications for eqn (57). First, the Clausius–Mossotti relation and conduction limit upper bound show the polarizability of a condensed phase (*e.g.*, liquid or solid) is approximately proportional to its volume. Here, 〈*r*^3^〉 is a proxy for the volume of an atom, so the sum of 〈*r*^3^〉 moments for atoms in a material is a proxy for the material's volume. The Clausius–Mossotti relation and conduction limit upper bound show the polarizability-to-volume ratio of a material is not strongly element-dependent and has modest dependence on the material's dielectric constant (see eqn (75) and (76)). Second, in condensed phases electrons undergo chemical potential equilibration that transfers some electron density from the least electronegative elements to the most electronegative elements. Accordingly, the chemical potential of the equilibrated condensed phase is not as extreme as either the most electropositive elements nor the most electronegative elements. For atoms in condensed materials, the polarizability to 〈*r*^3^〉 moment ratios will typically be lower than an isolated neutral alkali metal atom (extremely electropositive) on the one hand and higher than an isolated neutral fluorine atom (extremely electronegative) on the other hand. The polarizability to 〈*r*^3^〉 moment ratios of atoms in condensed materials thus exhibit a narrower range of values than for isolated atoms. Group 14 elements have approximately equal tendency to gain or lose electrons, thereby readily forming both positive and negative oxidation states. The isolated atom polarizability to 〈*r*^3^〉 moment ratios of Group 14 elements are approximately independent of the periodic row: C (0.34), Si (0.37), Ge (0.34), Sn (0.32), and Pb (0.31). Accordingly, we expect the same constant C in eqn (57) will work for both light and heavy elements.

Tests on the polarizabilities of 28 solids were performed to optimize C. The criteria are that without any upper bound imposed, the MRE should be 0–10% and after the upper bound is imposed, the MARE should be ≤∼10%. These criteria make sure the scaling law is as accurate as possible without the upper bound and the upper bound will not decrease the accuracy. Table 4 shows that *C* = 0.4 is optimal. This value is slightly higher than the polarizability to 〈*r*^3^〉 moment ratios of isolated Group 14 atoms.

**Table:** Comparison of the % error in the polarizability of 28 solids as a function of *C* value. “NU” means no upper bound is applied and “U” means the upper bound is applied

	*C* = 0.35	*C* = 0.4	*C* = 0.45	*C* = 0.35	*C* = 0.4
	NU	NU	NU	U	U
MRE	1.14%	6.87%	11.91%	−12.22%	−9.14%
MARE	26.75%	27.26%	29.09%	13.59%	11.89%

The characteristic frequency wp also has different expressions for isolated and buried atoms. For an isolated atom (*i.e.*, *m* = 1), wp should be proportional to *α*^−1/2^ which has been captured by the isolated atom scaling law. For a buried atom (*i.e.*, *m* ≪ 1), the polarizability becomes nearly proportional to 〈*r*^3^〉^AIM^ and C_6_ remains proportional to the atom's effective radius to the sixth power; therefore, wp is less sensitive to changes in *α* when the atom is buried. So the scaling for wp has been modified to:58
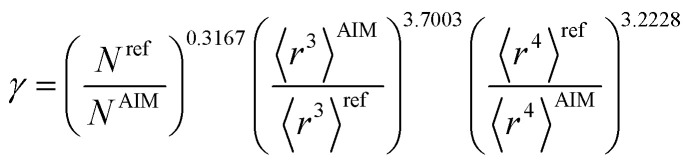
59
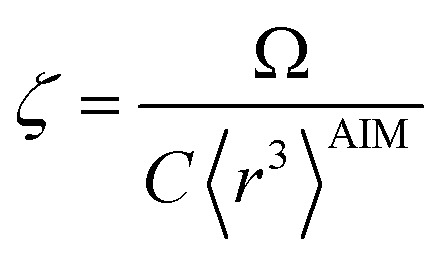

60 wp = *ζ*^(1−*m*^2^)/4^*γ*wp^ref^

when *m* = 1, eqn (60) reduces to the isolated atom scaling law in Section 3. A complete explanation and derivation of eqn (60) is given in Section S3 of ESI.†

### 4.4 Iterative polarizability screening

The non-directional, fluctuating, and induced dipole interaction tensors are defined in eqn (61)–(63):61
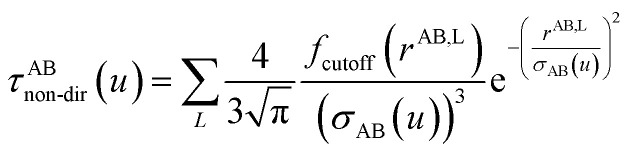
62
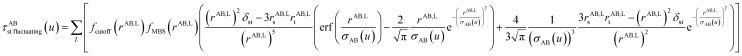
63
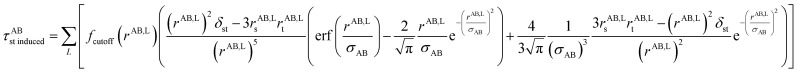
the subscripts s and t refer to spatial indices *x*, *y*, and *z*. This enables the non-directional and directional components to be separately screened. As described in Section 4.2, the conduction limit upper bound applies to the non-directionally screened (not the directionally screened) components. Computing static polarizabilities (*i.e.*, system response to constant externally applied electric field) requires directionally screened polarizabilities. As discussed in Section 4.7, parameterizing polarizable force fields requires non-directionally screened polarizabilities.

*f*_cutoff_(*d*_Ab_) is a smooth cutoff function that smoothly turns off dipole–dipole interactions between atoms as the distance between them increases:64
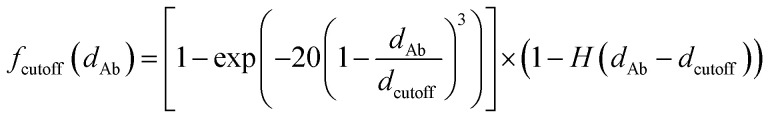
where *d*_Ab_ = *r*^AB,L^ is the distance between atom A and the image b in bohr. *d*_cutoff_ is the dipole interaction cutoff length. *H* is the Heaviside step function. As shown in Fig. S1 of ESI,† this function smoothly decreases from ∼1 at *d*_Ab_ = 0 to zero when *d*_Ab_ ≥ *d*_cutoff_. The power of three in the exponential ensures that both the first and second derivatives are continuous at *d*_Ab_ = *d*_cutoff_, which is required for frequency calculations. The factor of 20 in the exponent provides a balanced cutoff function steepness. Specifically, *f*_cutoff_(*d*_Ab_ ≤ 0.5 *d*_cutoff_) ≥ 0.9179 ensuring that all positions within half the cutoff distance are counted at nearly full strength.

Imagine a parameter 0 ≤ *λ* ≤ 1 that continuously turns on a particular screening type (*e.g.*, non-directional, fluctuating, or static). When *λ* = 0, the corresponding screening type is fully off. When *λ* = 1, the corresponding screening type is fully on. Thus, the corresponding screening process can be envisioned as transitioning continuously from *λ* = 0 to *λ* = 1. We expect the partially screened system (*i.e.* 0 < *λ* < 1) to have a polarizability intermediate between that for *λ* = 0 and *λ* = 1. Since the Gaussian width *σ*_AB_(*u*) depends on the polarizability (eqn (19)), the MCLF method continuously updates *σ*_AB_(*u*) during the screening process. In contrast, the TS-SCS method uses *σ*_AB_(*u*) corresponding to *λ* = 0 for the entire screening process, which does not allow the Gaussian width to update during the screening process.

The screening of each tensor is now an iterative process. In each iteration, we compute

65 *Q*_*j*+1_ = *D*_*j*_ + Δ_*j*_*τ*_*j*_

where Δ_*j*_ is the screening increment. Then *P*_*j*+1_ is computed as the inverse of *Q*_*j*+1_. The screening process is divided into non-directional and directional screening. Directional screening has two separate parts: screening for the static polarizability and screening for the fluctuating polarizabilities. Dividing the screening process into these three parts allows us to compute three different types of polarizabilities having distinct uses: (a) force-field polarizabilities that are suitable inputs for polarizable force-fields, (b) fluctuating polarizabilities for computing C_6_ coefficients *via* the Casimir–Polder integral, and (c) static induced polarizabilities corresponding to a constant externally applied electric field. As described in Section 4.2, this division also allows a polarizability upper bound to be applied. These three parts of the screening process are summarized below.

#### Non-directional screening

For non-directional screening, both *D* and *τ* are Natoms by Natoms matrices. In the first iteration, *j* = 1 and *D* is constructed using (*α*^unscreened^(*u*))^−1^ for the respective atoms along the diagonal and zeros elsewhere, and *σ*_AB_(*u*) is calculated from *α*^unscreened^(*u*). *τ*_j_ is defined in eqn (61) where *σ*_AB_(*u*) is computed using *P*_A,*j*_ and *P*_B,*j*_ in each iteration *j*,66
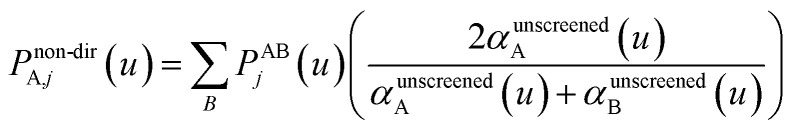
*D*_*j*_ is the matrix which has (*P*^non-dir^_A,*j*_(*u*))^−1^ of each atom on the diagonal and the rest is zero. The new *D*_*j*_ and *τ*_*j*_ are fed into eqn (65) for the next iteration. The calculation continues until67
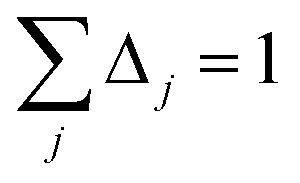
On the last iteration, *α*^raw_non-dir^_A_(*u*) is calculated *via*eqn (45).

Because the polarizability upper bound is calculated for uniform applied electric field and does not include any directional interactions between dipoles, it is applied at the end of non-directional screening and before directional screening. For each *u* value, the polarizability upper bound was applied to the raw non-directionally screened polarizability (*i.e.*, *α*^raw_non-dir^_A_(*u*)) *via* the following equation to generate68
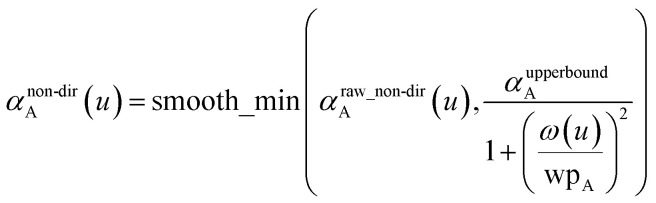
note that

69 *α*^force-field^_A_ = *α*^non-dir^_A_(*u* = Nimfreqs)

#### Directional screening for static polarizability

This procedure is only done at zero frequency. For directional screening, *D* and *τ* (eqn (63)) are 3Natoms by 3Natoms matrices. *D* is a block diagonal matrix which has the inverse polarizability tensor of each atom on the block diagonal and the rest is zero. In the first iteration, *D* and *σ*_AB_ are obtained using *α*^force-field^_A_. *P*_*j*_ undergoes a partitioned partial contraction (eqn (47)) to obtain partially screened polarizability tensors for each atom. The isotropic polarizability of each atom is 1/3 of the trace of their tensors. From this partially screened polarizability, the associated Gaussian screening width (eqn (19)) is obtained and fed into *τ*_*j*_. The polarizability tensor of each atom is inverted to construct a new *D* matrix. The new *D*_*j*_ and *τ*_*j*_ are fed into eqn (65) for the next iteration. The calculation continues until Δ_*j*_ sums to 1. The anisotropic polarizability correction is then applied as described in the next section.

#### Directional screening for fluctuating polarizabilities

*D* and *τ* (eqn (62)) are 3Natoms by 3Natoms matrices. *D* is a block diagonal matrix which has the inverse polarizability tensor of each atom on the block diagonal and the rest is zero. In the first iteration, *D* and *σ*_AB_(*u*) are calculated using *α*^non-dir^_A_(*u*). *P*_*j*_ undergoes a partitioned partial contraction (eqn (46)) to obtain partially screened polarizability tensors for each atom. The isotropic polarizability of each atom is 1/3 of the trace of their tensors. From this partially screened polarizability, the associated Gaussian screening width (eqn (19)) is obtained and fed into *τ*_*j*_. The polarizability tensor of each atom is inverted to construct a new *D* matrix. The new *D*_*j*_ and *τ*_*j*_ are fed into eqn (65) for the next iteration. The calculation continues until Δ_*j*_ sums to 1. On the last iteration, *α*^screened^_A_(*u*) is calculated *via*eqn (46). This procedure is done at multiple frequencies and the resulting {*α*^screened^_A_(*u*)} are fed into the Casimir–Polder integral to calculate the C_6_ dispersion coefficient of each atom. Note that

70 *α*^low_freq^_A_ = *α*^screened^_A_(*u* = Nimfreqs)

### 4.5 Anisotropic polarizability correction

We found that both the TS-SCS and MCLF screening processes often overestimate anisotropy of the static polarizability tensor. Physically, this occurs because the TS-SCS and MCLF methods use spherical Gaussian dipoles in their screening models. In reality, the effective screening width should be larger along the direction of increased polarizability and smaller along the direction of decreased polarizability. Consequently, using a spherical Gaussian dipole model under-screens along the direction of increased polarizability and over-screens along the direction of decreased polarizability. This inflates the polarizability tensor anisotropy.

This can be approximately corrected by mixing the pre-corrected AIM static polarizability tensor, 
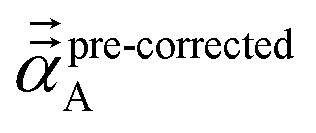
, with the isotropic AIM static polarizability, *α*^static^_A_:71
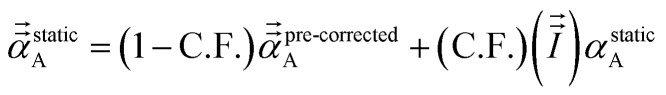
72
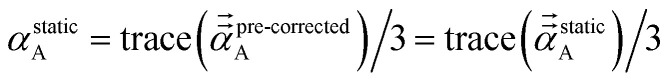
where the correction factor (C.F.) is between 0 and 1. Because the MCLF AIM static polarizability tensors have all non-negative eigenvalues, it follows from eqn (71) that the projection of 
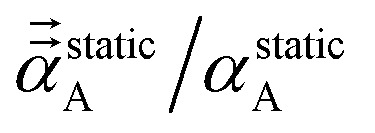
 onto any direction exceeds C.F.

To find a reasonable value for C.F., polarizability anisotropy ratios for four polyacenes were compared in Table 5. The reference ratios were calculated from Jiemchooroj *et al.*’s TD-DFT results.^78^ As shown in Table 5, C.F. = 0.2 gave the lowest mean relative error (MRE) and mean absolute relative error (MARE) for these materials. This value performed better than C.F. = 0 and better than the TS-SCS method. Therefore, we used C.F. = 0.2 as the regular value for the MCLF method. Except where otherwise specified, all reported MCLF results used this C.F. value.

Polarizability anisotropy ratios for four polyacenes. TS-SCS and MCLF (with various C.F. values) are compared to reference TD-DFT results

**Table:** 

	Reference			TS-SCS			C.F. = 0		
	*α*_*xx*_/*α*	*α*_*yy*_/*α*	*α*_*zz*_/*α*	*α*_*xx*_/*α*	*α*_*yy*_/*α*	*α*_*zz*_/*α*	*α*_*xx*_/*α*	*α*_*yy*_/*α*	*α*_*zz*_/*α*
C_6_H_6_	1.18	1.18	0.64	1.27	1.27	0.47	1.29	1.28	0.43
C_10_H_8_	1.42	1.04	0.54	1.49	1.10	0.41	1.52	1.10	0.38
C_14_H_10_	1.61	0.92	0.47	1.66	0.97	0.37	1.69	0.97	0.34
C_18_H_12_	1.76	0.83	0.41	1.78	0.88	0.34	1.82	0.87	0.31
MRE				−4.12%			−5.48%		
MARE				11.0%			13.8%

**Table:** 

	C.F. = 0.1			MCLF C.F. = 0.2			C.F. = 0.3		
	*α*_*xx*_/*α*	*α*_*yy*_/*α*	*α*_*zz*_/*α*	*α*_*xx*_/*α*	*α*_*yy*_/*α*	*α*_*zz*_/*α*	*α*_*xx*_/*α*	*α*_*yy*_/*α*	*α*_*zz*_/*α*
C_6_H_6_	1.26	1.26	0.49	1.23	1.23	0.54	1.20	1.20	0.60
C_10_H_8_	1.47	1.09	0.44	1.42	1.08	0.50	1.37	1.07	0.56
C_14_H_10_	1.62	0.97	0.40	1.55	0.98	0.47	1.48	0.98	0.54
C_18_H_12_	1.74	0.89	0.38	1.65	0.90	0.45	1.57	0.91	0.52
MRE	−2.57			0.35%			3.26		
MARE	8.31			5.66%			7.95		

As a second example, we chose a linear C_6_H_2_ molecule. This molecule's structure was optimized using B3LYP functional and def2QZVPPDD basis set in Gaussian09. Optimized bond lengths from one end to another were 1.06 (H–C), 1.21 (C–C), 1.35 (C–C), 1.21 (C–C), 1.35 (C–C), 1.21 (C–C), 1.06 (C–H) Å. The calculated molecular polarizability along the bonds is 181.15 au and perpendicular to the bonds is 41.83 au giving an isotropic polarizability of ⅔(41.83) + ⅓(181.15) = 88.27 au. The reference polarizability anisotropy ratios are 41.83/88.27 = 0.47 and 181.15/88.27 = 2.05 compared to 0.41 and 2.19 with C.F. = 0.2, and 0.26 and 2.48 with C.F. = 0.

Additional examples were provided by two test sets described in the following sections. For a test set containing 57 diatomic molecules (see Section 5.1), the MRE and MARE for eigenvalues are −6.90% and 11.77% with C.F. = 0.2, and −7.96% and 13.67% with C.F. = 0. For a test set containing small molecules (see Section 5.3), the MRE and MARE for eigenvalues are 5.45% and 8.10% with C.F. = 0.2, and 4.85% and 9.40% with C.F. = 0.

### 4.6 Multibody screening parameter (MBSP)

When a uniform external electric field is applied, the atomic dipoles induced by the field will align and atoms will interact with not only their neighbors but also atoms far away. The dispersion force, however, is caused by fluctuating dipoles. The fluctuating dipoles of the atoms will align with their neighbors but out of sync with atoms far away. The MBSP controls the length scale over which directional alignment persists:73
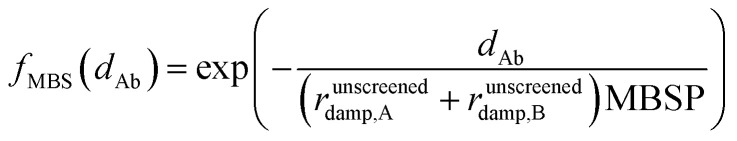
First-order exponential decay (eqn (73)) is the natural choice for the directional alignment function, because if 50% of the directional alignment persists over a distance *d*_half_, then over a distance 2 × *d*_half_ the expected persistence of directional alignment will be (50%) × (50%) = 25%. As defined in the ESI,†*r*^unscreened^_damp,A_ and *r*^unscreened^_damp,B_ are unscreened damping radii of the atoms-in-material. We optimized MBSP with the C_6_ of the 12 polyacenes and 6 fullerenes, the same set studied in Section 5.5. Table 6 shows the MRE and MARE of different MBSP values of which 2.5 is optimal.

**Table:** Comparison of the % error in C_6_ of polyacenes and fullerenes using different MBSP values

MBSP→		2.0	2.25	2.5	2.75
MRE	Polyacenes	−7.37%	−4.73%	−2.38%	−0.28%
	Fullerenes	5.16%	6.09%	6.84%	7.45%
MARE	Polyacenes	10.76%	9.14%	7.77%	7.01%
	Fullerenes	5.16%	6.09%	6.84%	7.45%

### 4.7 Flow diagram and explanation for three polarizability types

**Fig. 9:**
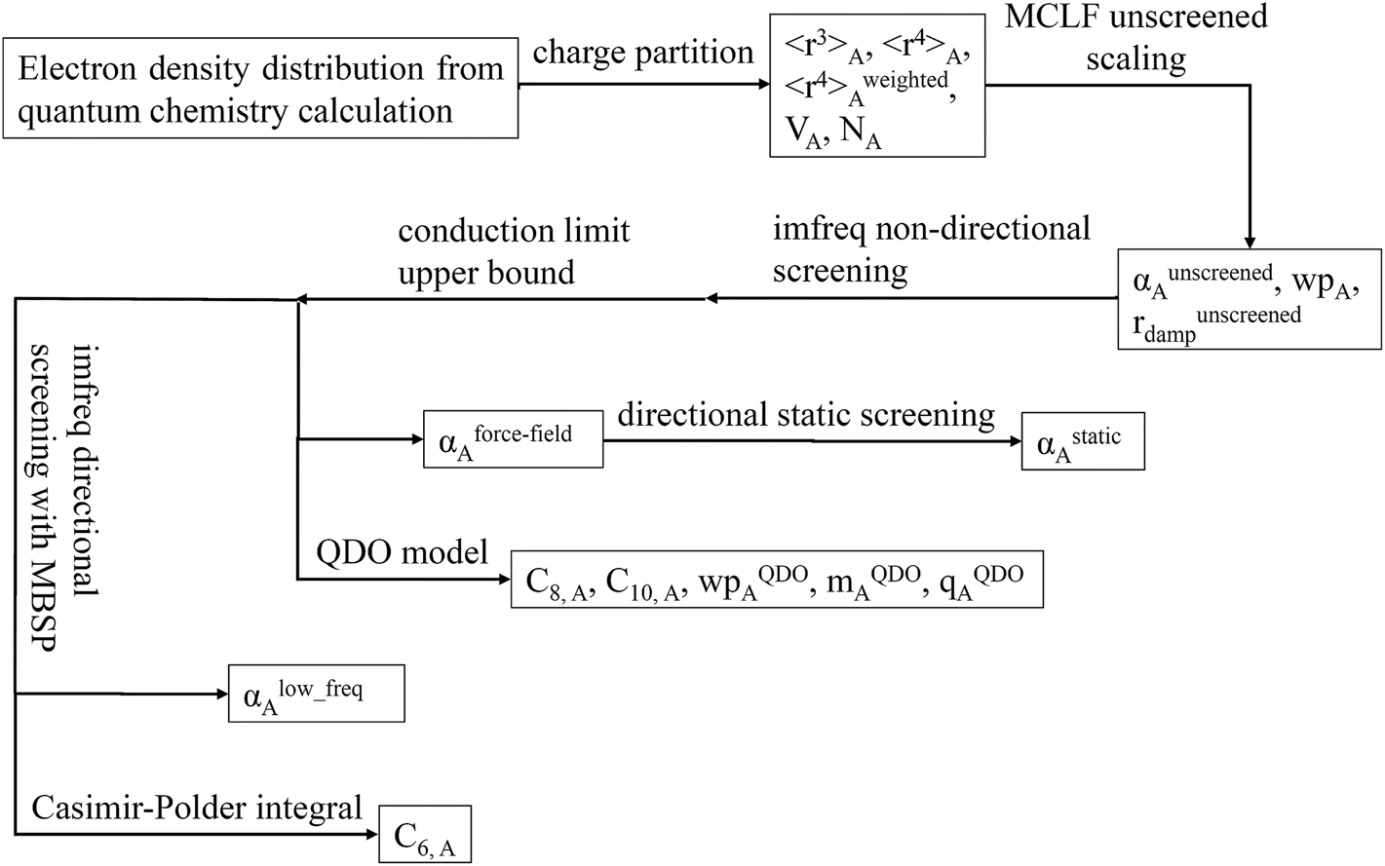
Flow diagram for MCLF method.

Fig. 9 is the flow diagram for MCLF method. This method yields three kinds of polarizabilities: *α*^force-field^, *α*^static^ and *α*^low_freq^. *α*^force-field^ is the polarizability with no directional ordering of the electric field and intended for use in force-field simulations. *α*^static^ is the static polarizability in a constant applied electric field. *α*^low_freq^ reflects the short-range order and long-range disorder of the fluctuating dipoles present in the dispersion interaction.

Because polarizability is a multibody effect, the polarizability of a molecule is not the sum of polarizabilities of isolated atoms. (In contrast to an atom in a material, the term isolated atom means an atom sitting alone in vacuum.) Directional interactions between atoms create components to the molecular polarizability that do not exist for the isolated atoms.^31,32^ Directional dipole–dipole interactions^31,32^ are considered during classical atomistic (*e.g.*, molecular dynamics or Monte Carlo) simulations that utilize polarizable force fields.^16,95^ To avoid double counting these directional dipole–dipole interactions, the force field's atomic polarizability parameters must be the non-directionally screened values.^96–99^ Many authors proposed direct additive partitioning of the quantum-mechanically computed molecular polarizability tensor into constituent atoms.^10,100–105^ Because those values already incorporate directional dipole–dipole interactions, their use in force fields would result in double counting the directional dipole–dipole interactions; therefore, we do not recommend their use as force field parameters. Our method provides both the non-directionally screened and directionally screened AIM polarizabilities, and the non-directionally screened values should be used as polarizable force-field parameters. The directional screening then arises during the course of the classical atomistic simulation.

Although the mixed C_6,AB_ dispersion coefficients could in principle be computed directly from the Casimir–Polder integral using the AIM polarizabilities at imfreqs, this would involve many integrations for materials containing thousands of atoms in the unit cell. Therefore, we used the following mixing formula which is consistent with both Padé approximation^81^ and QDO^9^ models:74
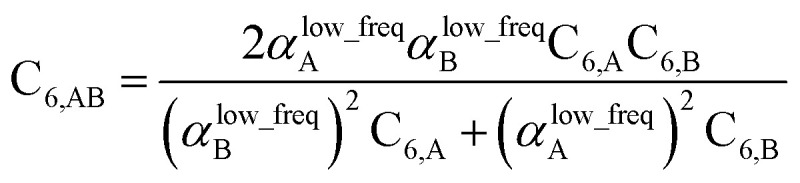
For MCLF, the polarizabilities appearing in eqn (74) must be *α*^low_freq^, because these are the polarizabilities associated with the dispersion interaction. Of note, the TS and TS-SCS methods use a similar mixing formula, except the polarizabilities appearing in the mixing formula are *α*^TS^ and *α*^TS-SCS^,^12,46^ because those methods do not yield *α*^low_freq^.

## 5. Results and discussion

Unless otherwise labeled, the following results are calculated using DDEC6 partitioning. The DDEC6 results were calculated using the Chargemol program.^61,66–68^ The MCLF results computed in this article avoided large matrix inversions. Details of the MCLF computational algorithm and computational parameters (*i.e.*, dipole interaction cutoff length, Rhomberg integration order, and Richardson extrapolation of the screening increments) are presented in the companion article.^79^ TS-SCS results in the present article were computed using matrix inversion *via* Gaussian elimination with partial pivoting (GEPP). The dipole interaction cutoff length for both MCLF and TS-SCS calculations was 50 bohr. Our MCLF calculations used a smooth cutoff function (eqn (64)). Our TS-SCS calculations used a hard cutoff (as consistent with prior literature^106^). As explained in the companion article, Rhomberg integration for both MCLF and TS-SCS used 16 imfreq points.^79^ For MCLF, Richardson extrapolation of the screening increments used the number of steps recommended in the companion article.^79^

### 5.1 Diatomic molecules

**Fig. 10:**
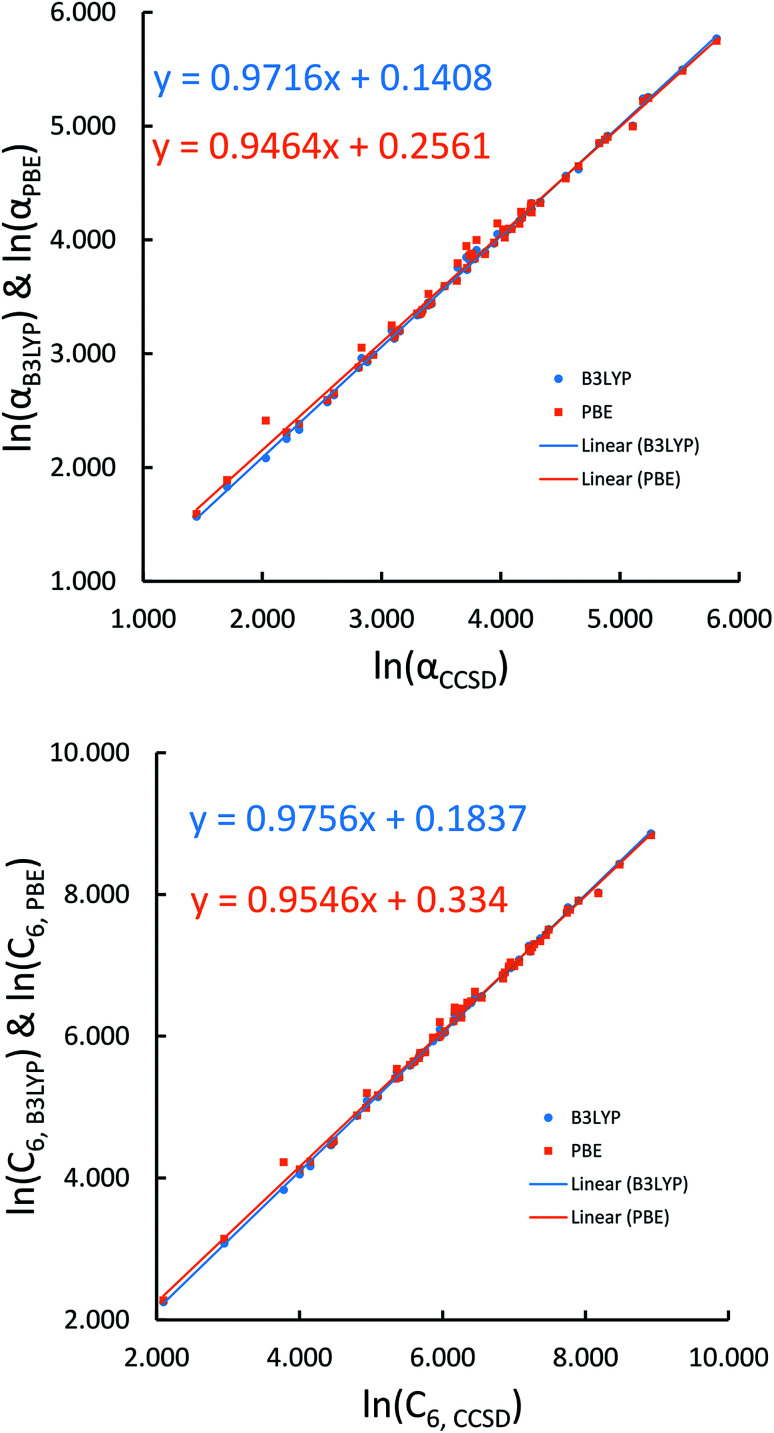
Comparison of MCLF *α* and C_6_ of 57 diatomic molecules computed using electron and spin density distributions from CCSD, PBE, and B3LYP calculations with large basis sets.

We first tested our model's sensitivity to the choice of exchange-correlation functional and basis set used to compute the system's electron and spin density distributions. A set of 57 diatomic molecules with elements across the periodic table were chosen as the test set. Three sets of electron density distributions of the test set were generated using Gaussian09 with CCSD/def2QZVPPDD, Gaussian09 with B3LYP/def2QZVPPDD, and VASP with PBE/planewave. The Chargemol program was then used to DDEC6 partition the electron density followed by MCLF analysis to obtain the static polarizabilities and C_6_ coefficients. Fig. 10 shows polarizability and C_6_ computed using CCSD/def2QZVPPDD densities *versus* polarizability and C_6_ computed using the PBE/planewave and B3LYP/def2QZVPPDD densities. This figure shows our model did not show strong dependence on the choice of exchange-correlation functional or basis set. Hence, MCLF gives similar results using electron density distributions from different proficient quantum chemistry levels of theory.

For the same 57 diatomic molecules, the isotropic static polarizability and the three eigenvalues of the static polarizability tensor from TS-SCS and MCLF were compared to the reference data. The reference is CCSD calculations with def2QZVPPDD basis set. System-specific polarizabilities for CCSD reference, TS-SCS, and MCLF are listed in the ESI.†Table 7 summarizes the error statistics. The TS-SCS method gave large errors independent of the charge partitioning method used (Hirshfeld or DDEC6). On average, MCLF was four times more accurate than TS-SCS for these materials.

**Table:** Comparison of the % error in the static polarizability of 57 diatomic molecules. The isotropic static polarizability equals one-third of the trace of the static polarizability tensor

	TS-SCS/H		TS-SCS/DDEC6		MCLF	
	Isotropic	Eigenvalues	Isotropic	Eigenvalues	Isotropic	Eigenvalues
MRE	34.4%	36.1%	27.8%	29.4%	−7.78%	−6.9%
MARE	41.1%	44.9%	36.6%	40.2%	10.4%	11.8%
Range	−21–437%	−35–484%	−24–440%	−35–491%	−34–17%	−40–49%

**Fig. 11:**
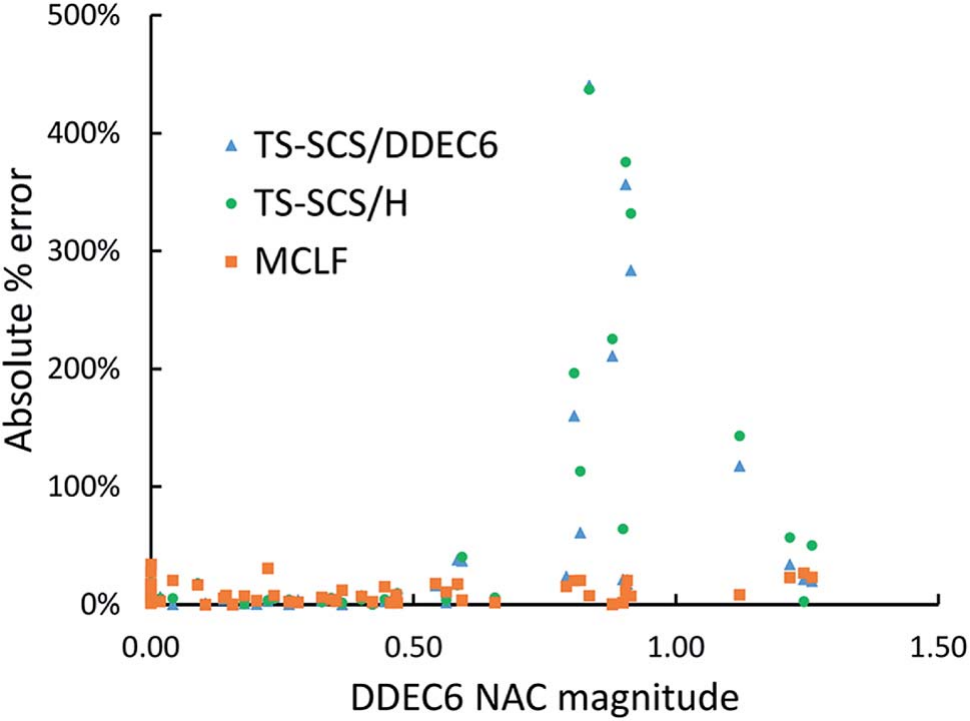
The absolute % error of isotropic static polarizabilities from TS-SCS/H, TS-SCS/DDEC6, and MCLF *versus* DDEC6 NAC magnitude of 57 diatomic molecules.

Fig. 11 is the absolute percentage error of isotropic polarizability from TS-SCS *versus* DDEC6 NAC magnitude for these diatomic molecules. From the plot, we can see TS-SCS gives large errors when the NAC magnitude is high. This confirms the TS-SCS model is less accurate for charged atoms and MCLF fixed this problem.

### 5.2 Dense periodic solids

Static polarizabilities were computed for 28 dense periodic solids including electric insulators, semi-conductors, and conductors. The geometries are from Inorganic Crystal Structure Database (ICSD).^107,110^ We generated electron densities in VASP^108^ using the PBE^109^ functional. See Gabaldon-Limas and Manz^68^ for a description of the VASP computational settings used.

Since the polarizability should not exceed the conduction limit, the reference static polarizability was set equal to the smaller value of the Clausius–Mosotti relation (eqn (75)) and conduction limit (eqn (76)):75
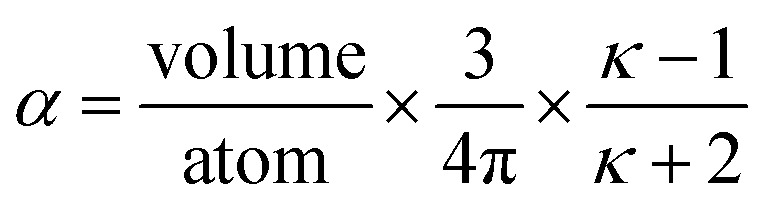
76
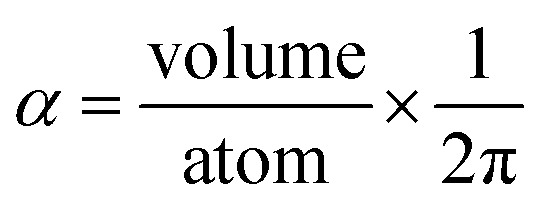
where volume is obtained from the ICSD^107,110^ crystal structure and *κ* is the experimental static dielectric constant. Results from different partitioning methods and screening methods were compared to this reference data. ICSD codes and computed results for individual materials are listed in the ESI.† As listed in the ESI,† the experimental dielectric constants were taken from Young and Frederikse^111^ and other sources.

Table 8 summarizes the error statistics. Comparing the MRE and MARE of the screened and unscreened polarizabilities with the same partitioning method (*e.g.*, TS/H *vs.* TS-SCS/H), screening increases the accuracy for each partitioning method. Because all of the unscreened methods gave >100% error for some materials, screening is an essential step in polarizability calculations. Comparing TS-SCS with different partitioning methods: IH is more accurate than H, and DDEC6 is more accurate than IH. The tendency of TS-SCS/H and TS-SCS/IH to overestimate polarizabilities for solids was previously reported by Bucko *et al.*^57^ with improved results reported using the fractionally ionic approach of Gould *et al.*^54^ (Those studies included some of the same materials as here.^54,57^) Using DDEC6 partitioning, MCLF gives more accurate results than TS-SCS with MARE of 12% and 24%, respectively.

Comparison of the % error in the computed static polarizabilities of 28 solids using different methods. H indicates Hirshfeld partitioning. IH indicates iterative Hirshfeld partitioning

**Table:** 

	Unscreened methods			
	TS/H	TS/IH	TS/DDEC6	Unscreened MCLF
MRE	297%	141%	86%	36%
MARE	297%	152%	93%	55%
Range	11–714%	−53–537%	−28–379%	−36–180%

**Table:** 

	Screened methods			
	TS-SCS/H	TS-SCS/IH	TS-SCS/DDEC6	MCLF
MRE	46%	16%	15%	−9%
MARE	47%	30%	24%	12%
Range	−10–76%	−53–50%	−31–50%	−37–14%

### 5.3 Polarizabilities of small molecules

A set of 22 molecules were selected from Thole^32^ and Applequist *et al.*^31^ that have experimentally measured isotropic static polarizability. Six of these also have experimentally measured polarizability tensor eigenvalues.^31,32^ The geometries are from geometry optimization we performed in Gaussian09 with B3LYP functional and def2QZVPPDD basis set. Tables 9 and 10 show that both TS-SCS/DDEC6 and MCLF performed well for this test set.

**Table:** Comparison of the isotropic static polarizability in atomic units for 22 molecules

	Reference	TS-SCS/DDEC6	MCLF		Reference	TS-SCS/DDEC6	MCLF
C_2_H_2_	22.472	23.540	27.855	CH_3_CN	30.233	33.818	34.111
C_2_H_4_	28.694	28.886	29.127	(CH_3_)_2_CO	43.122	47.864	43.785
H_2_O	9.785	9.168	9.845	CH_3_OCH_3_	35.361	38.102	36.270
C_6_H_6_	69.868	70.437	75.646	CH_2_ClCN	41.165	46.973	49.071
CF_4_	19.705	21.928	23.071	CH_2_OCH_2_	29.900	32.003	30.739
CFCl_3_	63.907	57.929	61.873	C_2_H_5_OH	34.282	37.546	35.429
NH_3_	16.129	13.862	14.368	H_2_CO	16.533	17.979	18.624
CO_2_	19.644	17.884	19.532	HCONH_2_	27.938	28.328	30.208
CS_2_	59.385	51.175	53.566	CH_2_Br_2_	60.735	57.041	57.983
C_3_H_8_	42.829	47.435	42.233	SF_6_	44.134	35.627	37.522
C_2_H_6_	30.233	32.626	29.131	SO_2_	26.993	25.164	26.650
MRE						1.04%	2.92%
MARE						8.74%	7.49%

**Table:** Comparison of the static polarizability tensor eigenvalues in atomic units for 6 molecules. For each molecule, the three eigenvalues were sorted smallest to largest

	Reference			TS-SCS/DDEC6			MCLF		
	Eigen 1	Eigen 2	Eigen 3	Eigen 1	Eigen 2	Eigen 3	Eigen 1	Eigen 2	Eigen 3
C_2_H_5_OH	30.368	33.607	38.870	30.219	34.512	47.909	31.648	33.579	41.061
CF_4_	19.705	19.705	19.705	21.928	21.928	21.928	23.071	23.071	23.071
C_2_H_6_	27.668	27.668	35.361	28.210	28.210	41.457	27.822	27.823	31.749
CH_3_CN	25.981	25.981	38.735	23.995	23.996	53.463	25.01	25.01	52.312
(CH_3_)_2_CO	31.380	48.959	49.027	35.472	49.630	58.489	35.706	47.354	48.294
CH_3_OCH_3_	29.625	33.337	43.054	31.823	31.989	50.495	32.853	32.989	42.968
MRE					8.75%			5.45%	
MARE					10.95%			8.10%	

### 5.4 C_6_ coefficients of atom/molecule pairs

This test involves C_6_ coefficients for pairs of atoms and molecules studied by Tkatchenko and Scheffler.^12^ Our geometries are from geometry optimization we performed in Gaussian09 with B3LYP functional and def2QZVPPDD basis set.

**Fig. 12:**
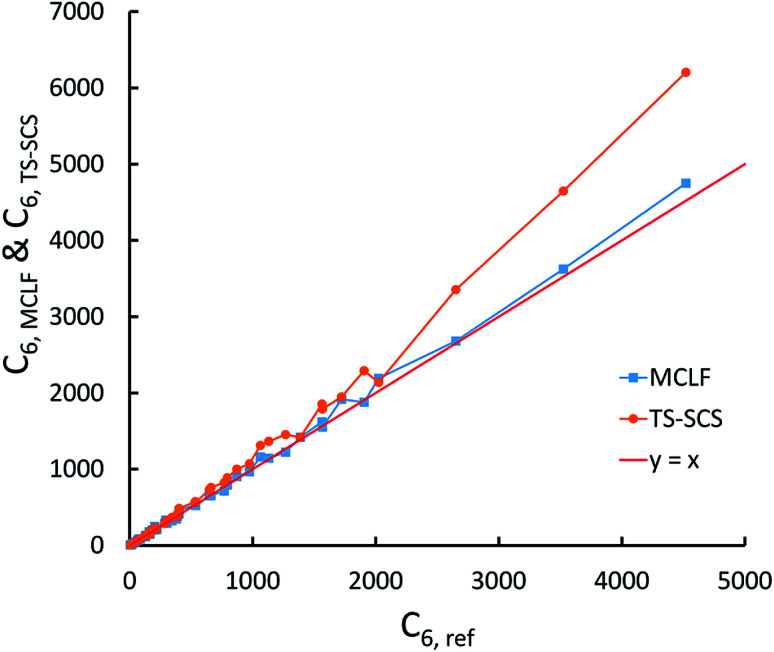
TS-SCS/DDEC6 and MCLF predicted C_6_ in atomic units for 49 atoms/molecules compared to experimentally-derived reference C_6_ values. TS-SCS/DDEC6 predicts too large C_6_ values for the larger molecules.

Fig. 12 compares the TS-SCS/DDEC6 and MCLF C_6_ coefficients to the reference values derived from the dipole oscillator strength distribution (DOSD) data of Meath and co-workers^74,75^ as tabulated by Bucko *et al.*^55^ As shown in Fig. 12, TS-SCS predicts too large C_6_ values for the larger molecules, while MCLF is consistently accurate for different sized molecules. For these 49 atoms/molecules, MCLF gave 1.15% MRE and 5.79% MARE while TS-SCS/DDEC6 gave 8.09% MRE and 10.29% MARE.

**Fig. 13:**
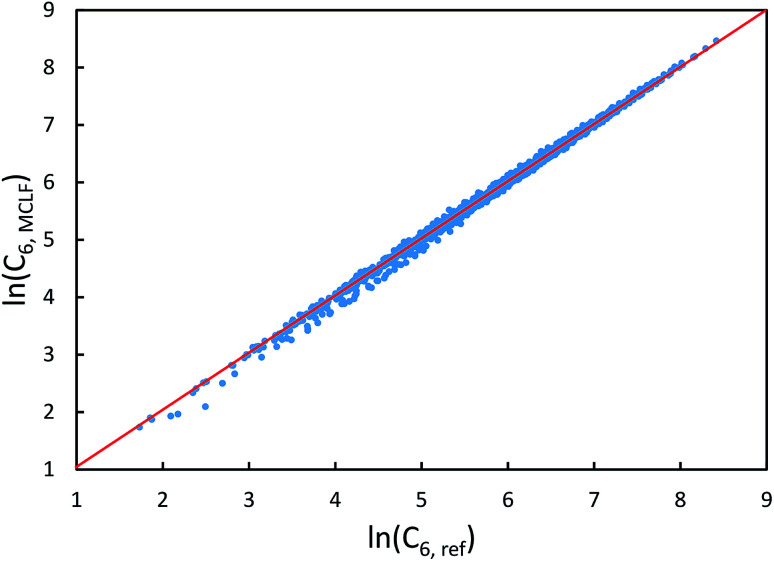
MCLF predicted C_6_ coefficients in atomic units for 1225 pairs formed from 49 atoms/molecules compared to the experimentally-derived reference C_6_ coefficients.

The same reference data source was also used for the 1225 pairs formed from these 49 atoms/molecules. Fig. 13 plots MCLF *versus* reference C_6_ values for these pairs. These MCLF C_6,AB_ values were computed from the C_6,A_ values using eqn (74). Then, the molecular C_6_ were computed from these C_6,AB_ using eqn (1). MCLF yielded highly accurate results with 0.80% MRE and 4.45% MARE. Results for individual materials in this data set are listed in the ESI.†

Compared to the dense solids discussed in Section 5.2 above, this test set is much less sensitive to the choice of charge partitioning method. For these same 1225 pairs, prior studies reported excellent results from the TS/H (MARE = 5.5%^12^ or 5.3%^55^), TS-SCS/H (MARE = 6.3%^46^), TS-SCS/IH (MARE = 8.6%^55^), D3 (MARE = 4.7%^112^), and D4 (MARE = 3.8%^37^) methods. The minimal basis iterative stockholder (MBIS) method at the TS/MBIS level gave a 6.9% root mean squared percent error for this test set (minus the Xe-containing dimers).^113^ Other studies also investigated this test set.

### 5.5 Polyacenes and fullerenes

Polycyclic aromatic compounds, such as polyacenes, and fullerenes, have strong directional alignment of induced and fluctuating dipoles in the ring planes. A set of 12 polyacenes and a set of 6 fullerenes were selected as test sets. We generated electron densities in VASP^108^ using the PBE^109^ functional. See Gabaldon-Limas and Manz^68^ for a description of the VASP computational settings used.

**Fig. 14:**
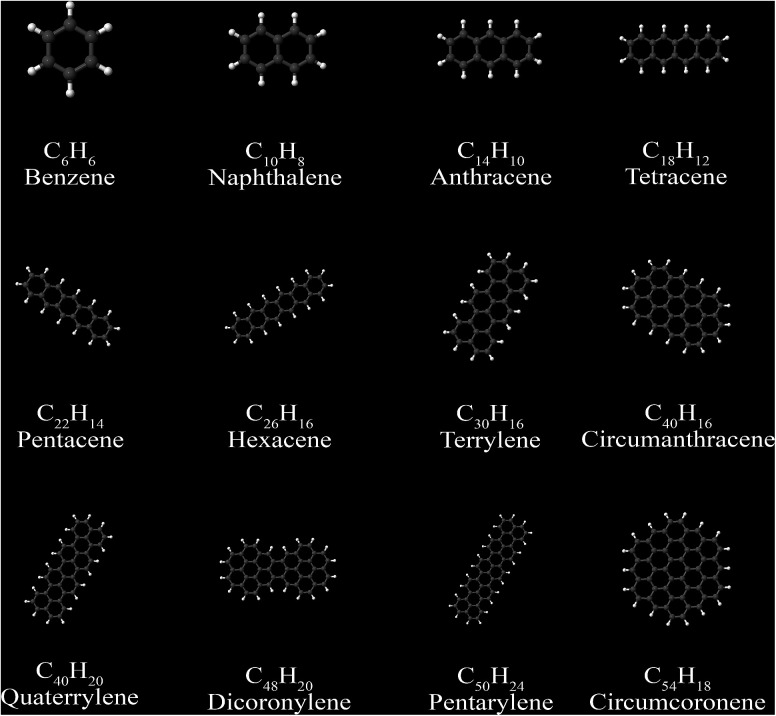
Structures of 12 polyacenes.

The polyacene geometries are from our VASP geometry optimization using the PBE functional. Polyacene reference static polarizabilities and C_6_ coefficients are from Marques *et al.*^77^ The polyacene structures are shown in Fig. 14. Computed results are summarized in Table 11. The MRE and MARE show MCLF was significantly more accurate than TS-SCS for describing both the static polarizabilities and the C_6_ coefficients of these materials.

**Table:** Comparison of the *α* and C_6_ in atomic units for 12 polyacenes

	Static polarizability, *α*			C_6_ dispersion coefficient		
	Reference	TS-SCS	MCLF	Reference	TS-SCS	MCLF
C_6_H_6_	70.5	71.864	79.100	1730	2018.50	1999.34
C_10_H_8_	123	122.500	133.052	4790	5641.58	5298.26
C_14_H_10_	189	181.475	197.738	9920	11 866.54	10 477.15
C_18_H_12_	264	247.574	271.016	17 500	21 191.56	17 595.20
C_22_H_14_	353	319.109	350.487	28 100	33 936.60	26 638.47
C_26_H_16_	454	395.191	434.779	42 100	50 407.20	37 626.79
C_30_H_16_	484	402.755	441.127	47 800	55 318.93	46 503.57
C_40_H_16_	612	523.270	590.151	82 500	95 014.04	81 069.67
C_40_H_20_	799	570.497	626.426	97 000	105 993.06	83 472.16
C_48_H_20_	770	665.562	745.500	122 000	147 407.13	118 431.09
C_50_H_24_	1196	748.553	822.271	168 000	175 992.78	131 300.24
C_54_H_18_	840	707.953	806.519	150 000	173 114.17	147 130.26
MRE		−13.15%	−4.14%		16.40%	−2.38%
MARE		13.47%	8.75%		16.40%	7.77%

Table 12 summarizes calculation results for fullerenes. The fullerene geometries are from Saidi and Norman.^114^ Tao *et al.* studied this set of fullerenes in 2016 and obtained excellent results using a hollow sphere model with modified single frequency approximation.^115^ The reference static polarizabilities and C_6_ coefficients are from Kauczor *et al.*’s TD-DFT calculations.^116^ For this test set, TS-SCS systematically underestimates the polarizabilities by 18.7% and C_6_ coefficients by 10.4%.

**Table:** Comparison of the *α* and C_6_ in atomic units for 6 fullerenes

	Static polarizability, *α*			C_6_ dispersion coefficient		
	Reference	TS-SCS	MCLF	Reference	TS-SCS	MCLF
C_60_	536.6	446.9	512.6	100 100	88 860	106 808
C_70_	659.1	534.4	616.6	141 600	125 502	150 150
C_78_	748.3	605.6	701.3	178 200	159 842	19 0785
C_80_	798.8	626.6	727.1	192 500	170 229	201 557
C_82_	779.7	642.9	746.1	196 800	179 254	213 111
C_84_	806.1	659.3	765.4	207 700	188 430	224 797
MRE		−18.7%	−5.92%		−10.4%	6.84%
MARE		18.7%	5.92%		10.4%	6.84%

In these two test sets, MCLF has better overall performance than TS-SCS with all four MCLF MAREs under 10% compared to all four TS-SCS MAREs over 10%. In contrast to TS-SCS, MCLF uses (i) an iterative update of the Gaussian dipole width and (ii) a multi-body screening function (eqn (73)) to describe decay of the fluctuating dipole directional order. These allow MCLF to describe induced and fluctuating dipole directional alignment effects more accurately than TS-SCS.

### 5.6 Large biomolecule

Non-reactive molecular mechanics force fields are model potentials containing several different parameter types: dispersion–repulsion parameters (*e.g.*, Lennard-Jones, Buckingham, or other forms), point charges or model atomic charge distributions (*e.g.*, Gaussian, Slater), flexibility parameters, and (optionally) polarizabilities.^117,118^ These molecular mechanics force fields enable classical atomistic simulations, such as molecular dynamics or Monte Carlo, to be performed over larger distance and time scales than would be practical using quantum chemistry methods such as DFT.^118–120^ These atomistic simulations are useful to estimate thermodynamic ensemble properties (*e.g.*, density, vapor pressure, adsorption isotherms, *etc.*), transport properties (*e.g.*, diffusion coefficients, viscosity, *etc.*), and structures (*e.g.*, protein folding and other conformational changes).^118,119,121–124^

An approach often used is to classify atoms into types, where similar atoms share the same atom type and same force-field parameters.^125,126^ We will refer to these as Typed Force Fields (TFF). TFFs have been successively improved over the past few decades by refining their atom type definitions and parameter values to make them more accurate and robust.^127–129^ Today, several TFFs perform reasonably well on various organic molecules and some small inorganic molecules.^122,123,130–133^

There are still areas for further improving force fields, especially for systems containing high chemical bonding diversity and charged ions. Metal-containing systems are especially prone to high chemical bonding diversity. For example, metal–organic frameworks (MOFs) can contain dozens of different metal elements in a plethora of different bonding motifs.^134–136^ While some efforts have been made to define new atom types for MOFs,^137^ the high chemical diversity makes it difficult to completely parameterize force fields for all MOFs using atom types. Quantum-mechanically derived force-fields (QMDFFs) are ideal for modeling these systems, because QMDFFs do not require pre-defined atom types.^138,139^ Machine learning is a recent approach in which force-field parameterization is trained to a machine learning model using QM-derived parameters.^4,140,141^ Advantages of the machine learning approach include high automation, fast computation, and the ability to handle large chemical diversity without having to manually define atom types.^4,140,141^

Non-polarizable force fields often model charged ions with reduced effective charges, but this places artificial constraints on the simulation.^142^ To make the parameters more transferable between different chemical systems and compositions, the reduced effective charge model should be replaced with a polarizable force-field.^16,19,143–145^ Kiss and Baranyai concluded for water that “It is impossible to describe the vapor–liquid coexistence properties consistently with nonpolarizable models, even if their critical temperature is correct.”^15^ Hence, an automated method like MCLF to assign atom-in-material polarizabilities is extremely important for modeling materials containing ions.

Useful insights can be gained by comparing TFF parameters for an atom type to QM-derived ones. Where the TFF parameters and the QM-derived ones are in good agreement, this validates the parameterization. Conversely, where the TFF parameters and the QM-derived ones differ substantially, this indicates areas for further study to potentially refine the atom type definitions and/or parameter values. A multimodal distribution or wide range of QM-derived parameter values suggests when to divide atoms into multiple atom types. A narrow distribution of QM-derived parameter values that is substantially offset from the TFF parameter value can indicate a need to update the TFF parameter value. In such a way, these comparisons can produce force-field improvements.

In this section, we compare MCLF C_6_ atom-in-material dispersion coefficients and polarizabilities to OPLS and AMOEBA force-field parameters, respectively, for the Human Immunodeficiency Virus reverse-transcriptase (HIV-RT) enzyme complexed with an inhibitor molecule. HIV is a retrovirus with RNA genome.^146^ Retroviruses replicate in a host cell by using a reverse-transcriptase enzyme to transcribe the virus's RNA genome into the host cell's DNA that is subsequently replicated by the host.^146^ Therefore, inhibition of the reverse-transcriptase enzyme is a potential way to slow virus replication, which is extremely important for controlling disease caused by the virus.^146^

**Fig. 15:**
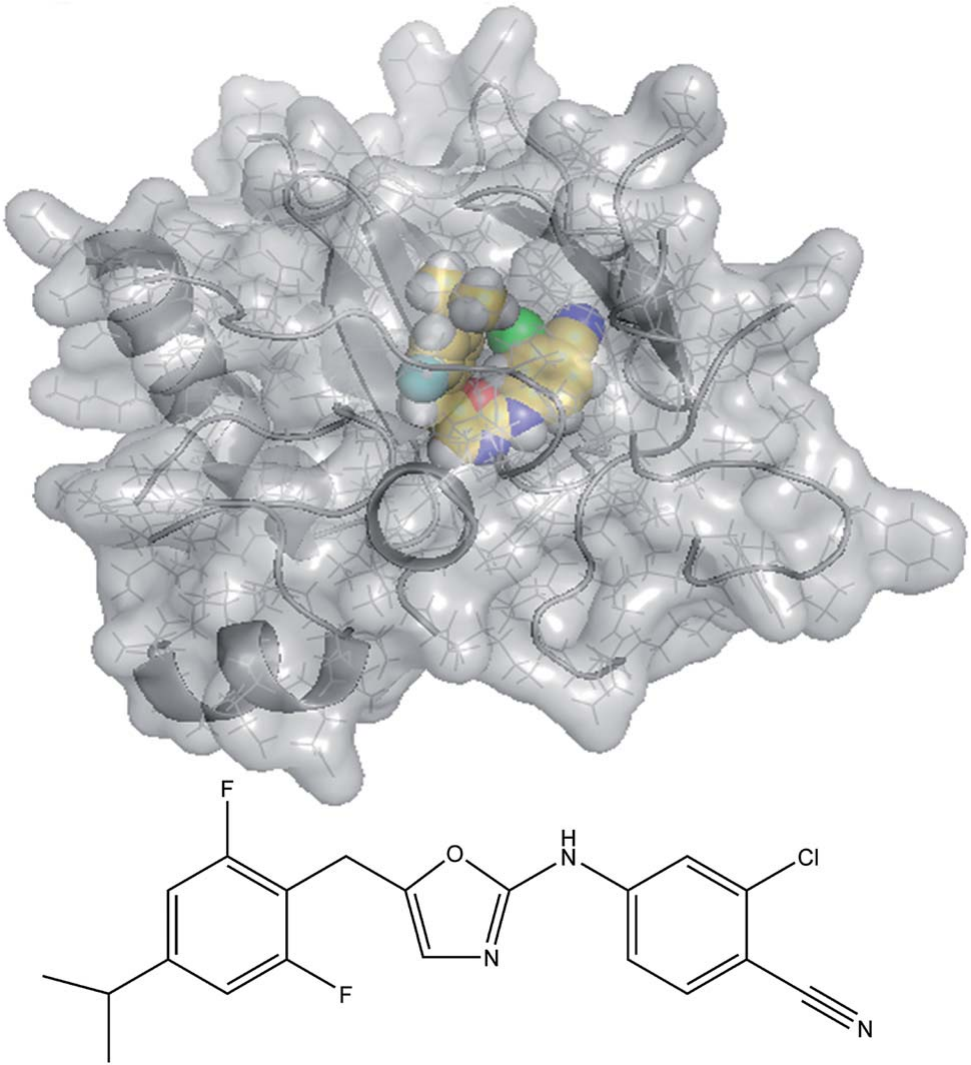
Structure of the inhibitor molecule and its complex with HIV reverse-transcriptase. In the complex, atoms of the inhibitor molecule are displayed as colored balls: yellow (C), white (H), red (O), blue (N), cyan (F), and Cl (green).

Bollini *et al.* and Cole *et al.* previously studied several oxazole derivatives as HIV-RT inhibitors.^147–149^ As an example of the MCLF method applied to macromolecules, we study one of these HIV-RT inhibitors, C_20_H_16_ClF_2_N_3_O (CAS # 1422256-80-1), shown in Fig. 15 together with a significant portion (2768 atoms) of its complex with wild-type HIV-RT. We computed the electron density distribution for this complex in ONETEP^150,151^ using the PBE^109^ exchange-correlation functional. Preparation of the input structure is described elsewhere.^147,148^ It was constructed from the 1S9E PDB file^152^ using the MCPRO^153^ and BOMB^154^ software. The 178 amino acids closest to the ligand were retained. The complex was solvated in a 25 Å water cap and equilibrated at room temperature for 40 million Monte Carlo steps using the MCPRO software. Water molecules were stripped from the final configuration, and the resulting structure (Fig. 15) was used as input for the ONETEP calculations.

Interactions between electrons and nuclei were described by Opium norm-conserving pseudopotentials.^155^ NGWFs were initialized as orbitals obtained from solving the Kohn–Sham equation for isolated atoms.^156^ The NGWFs were expanded as a psinc basis set^157^ with an equivalent plane-wave cutoff energy of approximately 1000 eV and the electron density was stored on a Cartesian grid of spacing 0.23 Bohr. The localization radii of the NGWFs were 10.0 Bohr. Calculations were performed using an implicit solvent model with a dielectric constant of 78.54 to mimic the water environment.^158^ Electron density partitioning was performed using the DDEC6 method implemented in the Chargemol program.^61,66–68^

Table 13 compares the computed C_6_ coefficients in the HIV-RT complex for a number of frequently-occurring OPLS atom types,^122^ including both backbone and side chain atoms. The C_6_ coefficients computed using MCLF generally show a much smaller range than the corresponding TS-SCS/DDEC6 data. Also, the OPLS force field C_6_ coefficients are usually closer to the MCLF results than to the TS-SCS results. While one should not draw too many conclusions from this, the OPLS parameters have been carefully fit over a number of decades to accurately reproduce experimental observables, such as organic liquid properties^122^ and protein NMR measurements.^159^ It is also noteworthy that the OPLS C_6_ coefficient is slightly higher than the MCLF result. This is expected since the C_6_ term in the OPLS force-field must effectively compensate for higher-order dispersion (C_8_, C_10_,…) terms that are not explicitly included in the OPLS force-field.^160^ The high value of C_6_ on the backbone carbonyl carbon (C (bb)) has been noted previously,^3^ and should be revisited in future force fields.

**Table:** Comparison of QM-derived and TFF C_6_ coefficients in atomic units for the HIV reverse transcriptase complex. Two QM-derived methods (MCLF and TS-SCS) are compared to the OPLS TFF. The mean unsigned deviation (MUD) quantifies the QM-derived C_6_ coefficient variation compared to the mean value for that atom type. bb signifies backbone atoms

Atom type	MCLF		TS-SCS		OPLS
	Range	Mean (MUD)	Range	Mean (MUD)	
N (bb)	31.7–47.2	39.0 (1.9)	33.7–71.9	48.2 (5.2)	58.2
H (bb)	0.7–1.2	0.9 (0.1)	0.2–1.4	0.6 (0.2)	0.0
C (bb)	20.1–28.9	24.4 (1.0)	19.3–54.8	34.7 (4.1)	84.8
O (bb)	25.3–39.9	31.8 (2.8)	18.4–31.5	24.1 (1.8)	41.0
CT (bb)	27.8–32.7	30.3 (0.9)	50.7–81.2	64.8 (5.0)	35.2
CT (CH3)	30.2–34.7	32.2 (0.8)	45.0–76.0	55.1 (4.2)	35.2
CT (CH)	24.7–37.0	30.5 (2.2)	35.7–98.8	53.8 (8.0)	35.2
HC	1.1–2.6	1.6 (0.2)	0.4–2.1	0.7 (0.2)	2.1
CA	32.6–43.1	36.9 (1.9)	36.0–65.2	45.7 (4.1)	40.7
HA	1.1–2.2	1.6 (0.2)	0.6–2.5	1.3 (0.3)	1.8

Table 14 compares the three kinds of MCLF polarizabilities (*i.e.*, force-field, static, and low_freq) to parameters used in the AMOEBA^16,144^ polarizable force field. For the H atoms, the AMOEBA polarizabilities are larger than the MCLF polarizabilities, though both are small compared to the polarizabilities of C, N, and O atoms. For the N (bb) atom, the AMOEBA polarizability is similar to the MCLF force-field polarizability. For the O (bb) atom, the AMOEBA polarizability (5.6) is smaller than the MCLF polarizability (6.9). Since the polarizability of an isolated neutral oxygen atom is ∼5.2,^65,80^ these polarizabilities are in line with O (bb) being slightly negatively charged and more diffuse than an isolated neutral oxygen atom. For the C atoms, the AMOEBA polarizabilities are substantially larger than the MCLF force-field polarizabilities and close to the MCLF static polarizabilities; this suggests the AMOEBA C atom polarizabilities include some directional screening effects. The AMOEBA polarizabilities use Thole^32^ model atomic charge distributions^16^*c*_1_ exp(−*c*_2_(*r*_A_)^3^) while the MCLF polarizabilities use Gaussian model charge distributions, so the optimized atomic polarizabilities can be different in these two methods and still approximately reproduce the molecular polarizability. Importantly, computed results presented earlier in this article show the MCLF force-field polarizabilities input into the dipole interaction tensor and solved yield good accuracy molecular static polarizabilities. Therefore, the MCLF force-field polarizabilities are appropriate for use in polarizable force-fields.

**Table:** Comparison of MCLF QM-derived and AMOEBA TFF polarizabilities in atomic units for the HIV reverse transcriptase complex. All three MCLF polarizabilities are listed: force-field, static, and low freq. The mean unsigned deviation (MUD) quantifies the MCLF polarizability variation compared to the mean value for that atom type. bb signifies backbone atoms

Atom type	MCLF (force-field)		MCLF (static)		MCLF (low freq)		AMOEBA
	Range	Mean (MUD)	Range	Mean (MUD)	Range	Mean (MUD)	
N (bb)	6.4–7.8	7.4 (0.2)	8.8–15.0	11.4 (0.9)	8.1–11.1	9.6 (0.4)	7.2
H (bb)	1.4–2.0	1.6 (0.1)	1.5–2.0	1.7 (0.1)	1.5–2.0	1.7 (0.1)	3.3
C (bb)	5.6–6.4	6.1 (0.1)	7.3–11.5	9.0 (0.5)	7.0–8.6	7.8 (0.2)	9.0
O (bb)	6.2–7.7	6.9 (0.3)	6.3–11.4	8.4 (0.9)	6.5–9.4	7.8 (0.5)	5.6
CT (bb)	6.7–7.3	7.0 (0.1)	8.8–11.3	9.9 (0.4)	8.1–9.0	8.5 (0.2)	9.0
CT (CH_3_)	7.0–8.0	7.6 (0.2)	8.3–10.1	9.0 (0.3)	8.0–8.9	8.4 (0.2)	9.0
CT (CH)	6.6–8.1	7.4 (0.3)	7.8–12.8	9.5 (0.7)	7.5–9.7	8.5 (0.4)	9.0
HC	1.6–2.9	2.1 (0.1)	1.7–3.1	2.2 (0.1)	1.6–3.0	2.1 (0.1)	3.3
CA	7.2–8.3	7.7 (0.2)	9.6–13.1	10.9 (0.6)	8.9–10.5	9.6 (0.3)	11.8
HA	1.8–2.4	2.0 (0.1)	1.9–2.9	2.3 (0.2)	1.9–2.6	2.2 (0.1)	4.7

OPLS atom types^122^ were assigned using MCPRO software^153^ and have the following descriptions. Sidechain atom types included: CT(CH_3_): alkane carbon bonded to three hydrogen atoms; CT(CH): alkane carbon atom bonded to one hydrogen atom; HC: alkane hydrogen atom; CA: carbon atom in an aromatic ring; HA: hydrogen bonded to an aromatic ring. Backbone atoms were classified according to: N: amide nitrogen; H: hydrogen bonded to amide nitrogen; C: amide carbon; O: amide oxygen; CT: alpha carbon atom. Only the most common atom types were analyzed to ensure adequate statistics. The substantial ranges of QM-derived parameter values in Tables 13 and 14 suggest there is room to further improve the atom type definitions. An alternative approach defines atom types as the set of unique local environment subgraphs that extend up to one or two bonds, and this approach has been successfully used with DDEC NACs.^4,68,161^ We recommend the atom type definitions be explored further in future work.

## 6. Conclusions

In summary, we introduced a new method (MCLF) to compute polarizabilities and dispersion force coefficients. This method can be applied to large and complex materials for which *ab initio* methods such as time-dependent DFT or CCSD perturbation response theory are too computationally expensive. Like TS-SCS, this method: (i) only requires the electron and spin density distributions as inputs, (ii) is capable of computing polarizability tensors and C_6_ coefficients for atoms-in-materials as well as for the whole molecule or unit cell, and (iii) works for materials containing 0, 1, 2, or 3 periodic boundary conditions. For TS-SCS, we showed that using DDEC6 partitioning increases accuracy compared to Hirshfeld and IH partitioning. The MCLF method achieves a long list of important improvements compared to existing methods:

(1) MCLF has new polarizability and C_6_ scaling relations for isolated atoms to set the reference values. These cover the charged atom states using a fundamentally different approach than the Fractional Ionic (FI) and TS-SCS methods. Unlike FI, this new approach does not require quantum mechanically computed reference polarizabilities and C_6_ values for isolated charged atoms; this is a huge advantage, because some isolated charged atoms are unstable. Unlike the TS method, this new approach describes changes in the polarizability-to-volume ratio with atomic charge state.

(2) MCLF uses a new polarizability partition and iterative polarizability screening to improve accuracy and avoid negative polarizabilities for highly charged atoms (*e.g.*, ZrO molecule) by partitioning the mixed pair contribution proportional to the polarizability of each atom in the pair. In contrast, the TS-SCS method sometimes assigns negative polarizabilities to atoms-in-materials, which may prohibit some subsequent calculations (*e.g.*, MBD or van der Waals radius) that require non-negative AIM polarizabilities as inputs. (A proof that *α*^force-field^, *α*^non-dir^(*u*), *α*^low_freq^, and *α*^screened^(*u*) are ≥0 is provided in the companion article.^79^)

(3) M scaling provides a unified scaling law describing the different behaviors of isolated atoms and buried atoms. This allows MCLF to accurately describe both surface and buried atoms. The TS-SCS method (which does not use m scaling) could not accurately describe static polarizabilities for polar diatomic molecules irrespective of the partitioning method.

(4) MCLF separates non-directional from directional screening of the dipole interaction tensor. The non-directionally screened polarizability is constrained to be less than or equal to the conduction limit upper bound, and this provides improved accuracy for buried atoms. In contrast, the TS-SCS method often produces atomic polarizabilities that are unphysically higher than the conduction limit.

(5) MCLF uses a multibody screening function to capture the fluctuating dipole alignment at short distances and disorder at long distances. This leads to more accurate C_6_ coefficients.

(6) MCLF computes three different types of screened dipole polarizabilities: (a) the non-directionally screened polarizabilities that are used as force-field and QDO input parameters, (b) the imfreq fluctuating polarizabilities that describe the local directional alignment contributing to C_6_ coefficients, and (c) the static polarizability containing long-range directional alignment of dipoles due to a constant externally applied electric field. MCLF computes the full polarizability tensors including diagonal and off-diagonal components.

(7) MCLF parameterizes a quantum Drude oscillator (QDO) model to yield higher-order (*e.g.*, quadrupolar and octupolar) AIM polarizabilities and higher-order AIM dispersion coefficients (*e.g.*, C_8_, C_9_, C_10_) and associated mixing rules.

(8) The MCLF atom-in-material polarizability tensors are always symmetric, while the TS-SCS atom-in-material polarizability tensors are sometimes asymmetric. Symmetric polarizability tensors are more convenient, because they can be displayed as ellipsoids.

(9) As explained in the companion article, the computational cost of MCLF scales linearly with increasing number of atoms in the unit cell for large systems.^79^ This is achieved using a dipole interaction cutoff function combined with computational routines that avoid both large matrix inversions and large dense matrix multiplications.^79^

Tests were performed on diverse material types: isolated atoms, diatomic molecules, periodic solids, small organic and inorganic molecules, fullerenes, polyacenes, and an HIV reverse transcriptase biomolecule. For each test set in this study, MCLF gave ≤12% MARE on the static polarizabilities, C_6_ coefficients, and static polarizability eigenvalues. This substantially improves over the TS-SCS method. For the static polarizabilities of solids: (a) TS-SCS with H, IH, and DDEC6 partitioning gave MARE of 47%, 30%, and 24%, respectively, (b) MCLF gave MARE of 12%, and (c) all of the unscreened methods gave much larger errors than the screened methods. For the static polarizabilities of diatomic molecules: (a) TS-SCS/H gave 41% MARE with a largest error of 437%, (b) TS-SCS/DDEC6 gave 37% MARE with a largest error of 440%, and (c) MCLF gave 10% MARE with a largest error of 34%. We anticipate MCLF should be useful for parameterizing polarizable force fields and DFT + dispersion methods.

There are several key differences between the MCLF and D3 and D4 approaches. The MCLF and D4 (ref. 35 and 37) approaches incorporate atomic charge information, while the D3 (ref. 11) method does not. The D3 and D4 approaches are based on the molecular geometry without requiring a quantum-mechanically computed electron density distribution,^11,35,37^ while MCLF uses a quantum-mechanically computed electron density distribution. (The atomic charges used in D4 method variants can be computed from a quantum-mechanically computed electron density distribution, but this is not required.^35,37^) The MCLF method explicitly considers dipole–dipole tensor interactions, while the D4 method only does so when a damped MBD Hamiltonian is included. (Without the MBD Hamiltonian, some dipole–dipole tensor interactions may be implicitly included in the D3 and D4 methods *via* coordination number effects, but only to the extent those dipole–dipole interactions correlate to the atom's coordination number and resemble dipole–dipole interactions in the reference compounds.) When using classical electronegativity equilibration NACs, the D4 method facilitates computing analytic forces during DFT + dispersion calculations^37^ MCLF yields AIM and system polarizability tensors, while D4 yields isotropic AIM and system polarizabilities. MCLF yields three types of polarizabilities: (a) non-directionally screened polarizabilities suitable for use in polarizable force-fields, (b) fluctuating polarizabilities that describe London dispersion interactions, and (c) static induced polarizabilities that include directional dipole–dipole interactions under a constant externally applied electric field.

As summarized in Table 15, we believe it is most useful to regard the MCLF method as a set of guiding principles and their empirical implementations. Some of the guiding principles are non-empirical (*i.e.*, derived from first-principles) while their implementation involves some empirical aspects (*i.e.*, fitting functional forms to observational data). Both the non-empirical and the empirical aspects of the MCLF method are highly innovative and important. Therefore, we do not believe it is reasonable to categorize the entire MCLF method as being exclusively empirical or exclusively non-empirical. If one had to choose a single word to categorize it, “semiempirical” is the appropriate choice. The Merriam-Webster dictionary defines semiempirical as “partly empirical”.^162^

**Table:** The MCLF method is a set of guiding principles and their empirical implementations. The first eight guiding principles are physically motivated. The last three guiding principles are motivated by computational efficiency

Guiding principle	Empirical implementation
Method should require reference polarizabilities and C_6_ coefficients for neutral free atoms only (not charged free atoms) and still properly describe scaling laws for charged atoms^a^	Isolated atom scaling laws (eqn (30) and (31))
Conduction limit upper bound for a material's non-directional polarizability at fixed geometry and fixed orientation	Conduction limit upper bound function (eqn (52)) applied to non-directional polarizabilities as a function of frequency (eqn (68)) using AIM volume (eqn (50))
Polarizability interaction not necessarily distributed equally between a pair of atoms	Proportional polarizability component partition (eqn (45)–(47))
Different polarizability scaling behaviors for surface and buried atoms	*m*-Scaling (eqn (53)–(60))
Fluctuating dipoles have a different effective range of directional alignment than static induced dipoles	Multi-body screening function (eqn (73)) used to compute *α*^screened^_A_(*u*)
Higher-order AIM polarizabilities and higher-order dispersion coefficients can be approximately described by a QDO model	QDO parameters computed from MCLF C_8,A_ (eqn (34)), C^non-dir^_6,A_, and *α*^force-field^_A_
*α* ^force-field^_A_ and *α*^screened^_A_(*u*) should be mathematically guaranteed to be non-negative	Iterative polarizability screening (see ref. 79 for proof)
Directional dipole–dipole interactions should not be double counted when using a polarizable force-field	*α* ^force-field^_A_ based on non-directionally screened dipole–dipole interactions
Use spherical Gaussian dipole model for computational simplicity followed by an anisotropic correction	Anisotropic polarizability correction (eqn (71))
For computational efficiency, there should be a simple mixing rule to compute C_6,AB_ from the individual AIM properties	Padé approximation (eqn (74))
Computational time and memory should scale linearly with increasing number of atoms in the unit cell for large systems	Dipole interaction cutoff function (eqn (64)), see ref. 79 for proof of linear scaling

a The physical basis for this is that some isolated anions have unbound electrons and therefore could not be used as reference states.

Notably, one could generate alternative empirical implementations of the same guiding principles. For example, the isolated atom scaling laws could be reconstructed using the 〈*r*^2^〉 and 〈*r*^4^〉 moments in place of the 〈*r*^3^〉 and 〈*r*^4^〉 moments, as shown in Table 1. This implementation change would usually produce minor differences in results, because both models are trained to the same underlying dataset of isolated atom polarizabilities and C_6_ coefficients. As another example, using a different but similar smooth minimum function to impose the conduction limit upper bound could usually produce similar results, because both functions are ultimately controlled by the same physical limit of the non-directional polarizability of an ideal conductor at fixed geometry and fixed orientation. Furthermore, the MCLF method must be applied using some chosen partitioning method (*e.g.*, DDEC6). Clearly, some partitioning methods are poor choices (*e.g.*, Hirshfeld as shown in Table 8), while others (*e.g.*, DDEC6) are good choices. The DDEC6 method is also a set of guiding principles (which contain some non-empirical aspects) and their empirical implementations that comprise a semiempirical method.^61,68^

## Conflicts of interest

There are no conflicts to declare.

## Supplementary Material

RA-009-C9RA03003D-s001

RA-009-C9RA03003D-s002
