# Supplementary material for: New scaling relations to compute atom-in-material polarizabilities and dispersion coefficients: part 1. Theory and accuracy
Source: RSC Adv. 2019 Jun 19;9(34):19297–324. doi: 10.1039/c9ra03003d (PMC9064874; doi:10.1039/c9ra03003d)
Supplement: RA-009-C9RA03003D-s002 [file RA-009-C9RA03003D-s002.pdf]

Electronic Supplementary Material (ESI) for RSC Advances.
This journal is © The Royal Society of Chemistry 2019

Electronic Supplementary Information for

New scaling relations to compute atom-in-material polarizabilities and dispersion coefficients: part 1. Theory and accuracy

Thomas A. Manz[1]*, Taoyi Chen[1], Daniel J. Cole[2], Nidia Gabaldon Limas[1], and Benjamin Fiszbein[1]

[1]Department of Chemical & Materials Engineering, New Mexico State University, Las Cruces, New Mexico, 88003-8001. [2]School of Natural and Environmental Sciences, Newcastle University, Newcastle upon Tyne NE1 7RU, UK

*Corresponding author email: tmanz@nmsu.edu

Contents

1. Damping radii definition

2. Def2QZVPPDD basis set definition

3. M scaling formula for wp

4. Plot of the smooth cutoff function

S1. Damping radii definition

The definition of damping radii in the discussion of this paper is the effective multibody screening radii. It is different from the radii that describe the location of energy minimum in the force field. The nominal interaction energy $C_6/\left(r_{damp}\right)^6$ should increase with increasing C_6. With this observation, a linear relation between $\ln(C_6)$ and $\ln(r_{damp})$ is possible. We developed the following relations to calculated the damping radii:

$$r_{damp}^{ref} = r_{vdW}^{NG}\left(\frac{C_6^{ref}}{C_6^{NG}}\right)^{1/9} \tag{S1}$$

$$r_{damp}^{unscreened} = r_{damp}^{ref}\left(\frac{C_6^{unscreened}}{C_6^{ref}}\right)^{1/9} \tag{S2}$$

The notation "ref" means the property of a free atom, "NG" means the property of the noble gas free atom in the same period of the periodic table of elements. r_{vdW}^{NG} is the van der Waals radius of the noble gas atoms from Tang and Toennies.[S1] Eqn (S1)–(S2) cause the nominal interaction energy $C_6/\left(r_{damp}\right)^6$ to vary as $\sim\left(C_6\right)^{1/3}$, thereby providing a smooth increase in the nominal interaction energy as C_6 increases.

The damping radii for elements 87–109 are calculated using extrapolated reference value for Og (element 118) using the following equations:

$$r_{vdW}^{Og} = \frac{\left(r_{vdW}^{Rn}\right)^2}{r_{vdW}^{Xe}} \tag{S3}$$

$$C_6^{Og} = \frac{\left(C_6^{Rn}\right)^2}{C_6^{Xe}} \tag{S4}$$

The assumption is that there are same number of elements from Xe to Rn and Rn to Og, hence the ratio of the C_6 and damping radii of Xe/Rn and Rn/Og should be roughly the same.

Although not used in any subsequent calculations presented in this article, a set of screened damping radii are also computed and printed:

$$r_{damp}^{screened} = r_{damp}^{unscreened}\left(\frac{\alpha^{low\_freq}}{\alpha^{unscreened}}\right)^{1/3} \tag{S5}$$

These screened atom damping radii may be useful to develop correlations for the non-bonded (e.g., Lennard-Jones or related) parameters used to construct force fields, but a further analysis is beyond the scope of the current article.

S2. Def2QZVPPDD basis set definition

The def2QZVPPDD basis set we used for CCSD calculations is modified from the def2QZVPPD basis set[S2] obtained from the EMSL Basis Set Exchange[S3, S4]. We added another set of diffuse functions to s, p, d, f functions by dividing the smallest α of the corresponding functions by 2.5. No diffuse functions were added to g. For example, in the basis set of Cl the smallest α for s basis functions is 0.054234565513, so the added diffuse function for s has α = 0.0216938262. For this same example, the smallest α for p basis functions is 0.027256146018, so

the added diffuse function for p has α = 0.0109024584. The same procedure is used for d and f but not g. For elements with relativistic effective core potential (RECP), def2QZVPPDD uses exactly the same RECP as def2QZVPPD. The enclosed def2QZVPPDD.gbs and def2QZVPPDD.pseudo files contain the full basis set and RECP parameter tabulations, respectively.

S3. M scaling formula for wp

Table S1 contains a dimensional analysis for our isolated atom scaling law. α, C_6, and wp scale approximately proportional to the isolated atom's effective radius raised to the 4, 6, and -2 powers, respectively.

Table S1: Dimensional analysis for our isolated atom scaling law. The number in each column is the effective exponent in the scaling law. The number in parentheses is the rounded exponent.

	N_{elec}	r
α	0.4607 (0.5)	3.8517 (4)
C_6	1.0823 (1)	5.9131 (6)
wp	0.1608 (0)	-1.7903 (-2)

In contrast, the polarizability of a buried atom scales approximately proportional to the atom's effective radius raised to the power 3. As explained in the main text, this polarizability scaling for buried atoms has its physical origins in the Clausius-Mossotti relation and conduction limit upper bound. Mathematically, our method imposes this change in radial moment power from surface to buried atom polarizability using m scaling. As explained in the main text, m = 1 for an isolated atom and $m \approx 0$ for a buried atom.

The wp scaling of a partially or fully buried atom can be inferred using analytic continuation from m = 1 to $0 < m < 1$. Imagine a process in which all inter-nuclear distances in the material are scaled by a factor λ . Starting with $\lambda \gg 1000$, the atoms in the material will be fully separated (i.e., m=1). We then imagine a hypothetical process in which λ is smoothly decreased until $\lambda = 1$ (i.e., the true material is recovered). This hypothetical process corresponds to smoothly changing from m = 1 (when $\lambda \gg 1000$) to $0 < m < 1$ (when $\lambda = 1$). By tracking the wp changes along this hypothetical path, we can recover the wp scaling law for the partially or fully buried atom.

For all atoms in materials, C_6 scales approximately proportional to the atom-in-material's effective radius raised to the power 6. Hence, C_6 scales approximately proportional α^2 for a buried atom. In contrast, C_6 scales approximately proportional to $\alpha^{3/2}$ for a free atom (see reference S5 and our isolated atom scaling law).

To track these wp changes, we must first relate them to the polarizability scaling. From $wp = 4C_6/(3\alpha^2)$ it follows that wp scales approximately proportional to $\alpha^{-1/2}$ for a free atom and nearly independent of α for a buried atom. For convenience, let us define τ as the coordinate (i.e., temporary value of m) along the continuous path from 1 to the true m. Then, we can expand wp as

$$wp = wp^{ref}\gamma \cdot \left(\left(\frac{\alpha(\tau_0 = 1)}{\alpha(\tau_1)} \right) \left(\frac{\alpha(\tau_1)}{\alpha(\tau_2)} \right)^{\tau_1} \left(\frac{\alpha(\tau_2)}{\alpha(\tau_3)} \right)^{\tau_2} \cdots \left(\frac{\alpha(m - d\tau)}{\alpha(m)} \right)^{m-d\tau} \right)^{1/2} \quad (S6)$$

where $wp^{ref}\gamma$ is the value of wp from the isolated atom scaling law (i.e., when m=1) and the path is described by $\tau_1 = 1 + d\tau$, $\tau_2 = 1 + 2d\tau$, $\tau_3 = 1 + 3d\tau$, ... to m. Representing eqn (S6) in logarithmic format gives

$$\frac{d\ln(wp)}{d\tau} = -\frac{\tau}{2}\frac{d\ln(\alpha)}{d\tau} \quad \text{(S7)}$$

When $\tau = 1$ (i.e., starting with isolated atom), changes in wp scale locally proportional to $\alpha^{-1/2}$. When $\tau = 0$ (e.g., ending with buried atom), then changes in wp scales locally independent of α. Between these two extremes, changes in wp scale locally proportional to $\alpha^{-\tau/2}$.

From the article's main text,

$$\frac{d\ln(\alpha)}{d\tau} = \ln\left(\frac{\Omega}{C\langle r^3\rangle^{AIM}}\right) = \ln(\zeta) \quad \text{(S8)}$$

Substituting eqn (S8) into (S7) and integrating gives

$$\int_{wp^{ref}\gamma}^{wp(final)} d\ln(wp) = \int_1^m -\frac{\tau}{2}\ln(\zeta)d\tau \quad \text{(S9)}$$

Integrating eqn (S9) yields

$$\ln\left(\frac{wp}{wp^{ref}\gamma}\right) = \left(\frac{1-m^2}{4}\right)\ln(\zeta) \quad \text{(S10)}$$

$$wp = \zeta^{(1-m^2)/4}\gamma wp^{ref} \quad \text{(S11)}$$

S4. Plot of the smooth cutoff function

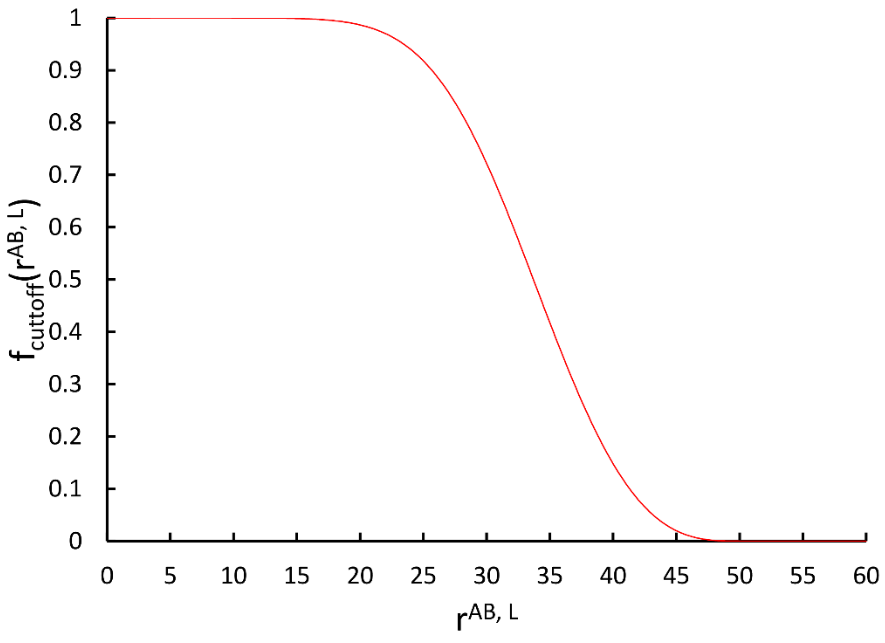

Figure S1: Plot of the smooth cutoff function controlling the dipole-dipole interactions. This function gives continuous first and second derivatives at the cutoff radius of 50 bohr.

References:

S1. K. T. Tang and J. P. Toennies, The van der Waals potentials between all the rare gas atoms from He to Rn, *J.Chem. Phys.*, 2003, **118**, 4976-4983. doi:10.1063/1.1543944

S2. D. Rappoport and F. Furche, Property-optimized Gaussian basis sets for molecular response calculations, *J. Chem. Phys.*, 2010, **133**, 134105. doi:10.1063/1.3484283

S3. D. Feller, The role of databases in support of computational chemistry calculations, *J. Comput. Chem.*, 1996, **17**, 1571-1586. doi:10.1002/(SICI)1096-987X(199610)17:13<1571::AID-JCC9>3.0.CO;2-P

S4. K. L. Schuchardt, B. T. Didier, T. Elsethagen, L. S. Sun, V. Gurumoorthi, J. Chase, J. Li and T. L. Windus, Basis Set Exchange: A community database for computational sciences, *J. Chem. Inf. Model.*, 2007, **47**, 1045-1052. doi:10.1021/ci600510j

S5. T. Gould and T. Bucko, C_6 coefficients and dipole polarizabilities for all atoms and many ions in rows 1−6 of the periodic table, *J. Chem. Theory Comput.*, 2016, **12**, 3603-3613. doi:10.1021/acs.jctc.6b00361
